# NEMF mutations that impair ribosome-associated quality control are associated with neuromuscular disease

**DOI:** 10.1038/s41467-020-18327-6

**Published:** 2020-09-15

**Authors:** Paige B. Martin, Yu Kigoshi-Tansho, Roger B. Sher, Gianina Ravenscroft, Jennifer E. Stauffer, Rajesh Kumar, Ryo Yonashiro, Tina Müller, Christopher Griffith, William Allen, Davut Pehlivan, Tamar Haral, Martin Zenker, Denise Howting, Denny Schanze, Eissa A. Faqeih, Naif A. M. Almontashiri, Reza Maroofian, Henry Houlden, Neda Mazaheri, Hamid Galehdari, Ganka Douglas, Jennifer E. Posey, Monique Ryan, James R. Lupski, Nigel G. Laing, Claudio A. P. Joazeiro, Gregory A. Cox

**Affiliations:** 1 The Jackson Laboratory, Bar Harbor, ME USA; 2 The University of Maine, Graduate School of Biomedical Science and Engineering, Orono, ME USA; 3 Center for Molecular Biology of Heidelberg University (ZMBH), DKFZ-ZMBH Alliance, Heidelberg, Germany; 4 Department of Neurobiology & Behavior, Stony Brook University, Stony Brook, NY USA; 5 Center for Nervous System Disorders, Stony Brook University, Stony Brook, NY USA; 6 Harry Perkins Institute of Medical Research, Centre for Medical Research, University of Western Australia, Nedlands, WA Australia; 7 Department of Molecular Medicine, Scripps Research, Jupiter, FL USA; 8 College of Medicine Pediatrics, University of South Florida, Tampa, FL USA; 9 Mission Fullerton Genetics Center, Mission Health, Asheville, NC USA; 10 Department of Molecular and Human Genetics, Baylor College of Medicine, Houston, TX USA; 11 Human Genome Sequencing Center, Baylor College of Medicine, Houston, TX USA; 12 Department of Genetic and Metabolic Diseases, Hadassah-Hebrew University Medical Center, Jerusalem, Israel; 13 Institute of Human Genetics, Otto-von-Guericke University Magdeburg, Magdeburg, Germany; 14 Department of Genetics, King Fahad Medical City, Riyadh, Saudi Arabia; 15 The Center for Genetics and Inherited Diseases, Taibah University, Almadinah Almunwarah, Saudi Arabia; 16 Faculty of Applied Medical Sciences, Taibah University, Almadinah Almunwarah, Saudi Arabia; 17 Neurogenetics Laboratory, UCL Queen Square Institute of Neurology, London, UK; 18 The National Hospital for Neurology and Neurosurgery, London, UK; 19 Department of Genetics, Faculty of Science, Shahid Chamran University of Ahvaz, Ahvaz, Iran; 20 GeneDx, Inc, Gaithsberg, MD USA; 21 Department of Neurology, The Royal Children’s Hospital, Melbourne, VIC Australia; 22 Murdoch Children’s Research Institute and University of Melbourne, Melbourne, VIC Australia; 23 Department of Pediatrics, Baylor College of Medicine, Houston, TX USA; 24 Texas Children’s Hospital, Houston, TX USA; 25 Department of Molecular Medicine, Scripps Research, La Jolla, CA USA

**Keywords:** Mechanisms of disease, Disease genetics, Neurodegeneration, Neuromuscular disease

## Abstract

A hallmark of neurodegeneration is defective protein quality control. The E3 ligase Listerin (LTN1/Ltn1) acts in a specialized protein quality control pathway—Ribosome-associated Quality Control (RQC)—by mediating proteolytic targeting of incomplete polypeptides produced by ribosome stalling, and *Ltn1* mutation leads to neurodegeneration in mice. Whether neurodegeneration results from defective RQC and whether defective RQC contributes to human disease have remained unknown. Here we show that three independently-generated mouse models with mutations in a different component of the RQC complex, NEMF/Rqc2, develop progressive motor neuron degeneration. Equivalent mutations in yeast Rqc2 selectively interfere with its ability to modify aberrant translation products with C-terminal tails which assist with RQC-mediated protein degradation, suggesting a pathomechanism. Finally, we identify *NEMF* mutations expected to interfere with function in patients from seven families presenting juvenile neuromuscular disease. These uncover NEMF’s role in translational homeostasis in the nervous system and implicate RQC dysfunction in causing neurodegeneration.

## Abstract (web summary)

Defective protein quality control is a key feature of neurodegeneration. Here, the authors show that mutations in *Nemf/NEMF*, a component of the Ribosome-associated Quality Control complex, have a neurodegenerative effect in mice and may underlie neuromuscular disease in seven unrelated families.

## Introduction

Ribosomes can stall during translation for a variety of reasons, such as the absence of termination codons followed by translation into the mRNA poly(A) tail (as in nonstop mRNA) or the deficiency of charged tRNAs^1,2^. Ribosome stalling is a critical problem, as it sequesters ribosomal subunits from the translation-competent pool and, in addition, gives rise to aberrant (incomplete) nascent polypeptide chains that are potentially toxic. In eukaryotes, stalled ribosomes are resolved by rescue factors that split the ribosomal subunits but leave the nascent chain still linked to tRNA, obstructing the exit tunnel in the 60S subunit^3–5^. This nascent chain-tRNA/60S aberrant structure is recognized by nuclear export mediator factor (NEMF; Rqc2 in yeast). NEMF/Rqc2 facilitates the recruitment of the E3 ligase LTN1 (Ltn1 in yeast), which in turn ubiquitinates the nascent chain, leading to its proteasomal degradation^6–12^. When ubiquitylation fails, Rqc2 additionally acts by catalyzing the C-terminal elongation of 60S-anchored nascent chains with untemplated Alanine and Threonine tails (CAT tails)^11^. An Rqc2 homolog was also found to catalyze C-terminal tailing in bacteria, where the modification acts as a direct proteolysis signal—a function that is likely to have evolved as early as in the Last Universal Common Ancestor^13^. In eukaryotes, the biological functions of CAT tails are not fully understood, however recent evidence suggests that they can assist Ltn1 by exposing Lys residues that may be buried in the ribosomal exit tunnel for ubiquitylation^14^.

We had previously reported that *Ltn1* mutation causes motor neuron degeneration in mice^15^. Whether neurodegeneration results from defective RQC and whether defective RQC also contributes to human disease remains unknown. The findings that Ltn1/LTN1 and Rqc2/NEMF work closely in RQC predicted that, if the phenotype of *Ltn1*-mutant mouse is a consequence of faulty RQC, defective NEMF function might have a similar effect. Accordingly, here we describe two novel mouse models with different N-ethyl N-nitrosourea (ENU)-induced missense mutations in *Nemf*, as well as a genetically engineered NEMF-deficient model, which exhibit neurodegeneration and motor deficits. Functional analyses show that the ENU-equivalent mutations lead to altered CAT tail synthesis (“CATylation”) by yeast Rqc2. Finally, we describe nine cases from seven unrelated families with neuromuscular phenotypes harboring *NEMF* variants. Thus, our results provide evidence that NEMF functions in neuronal homeostasis and that its loss causes neurodegeneration and motor phenotypes in mice and humans. These findings, along with the previously described *Ltn1* mouse model^15^, strongly point to RQC as a critical protein quality control pathway protecting neurons against degeneration.

## Results

### Mouse *Nemf* mutations result in progressive motor phenotypes

**Fig. 1:**
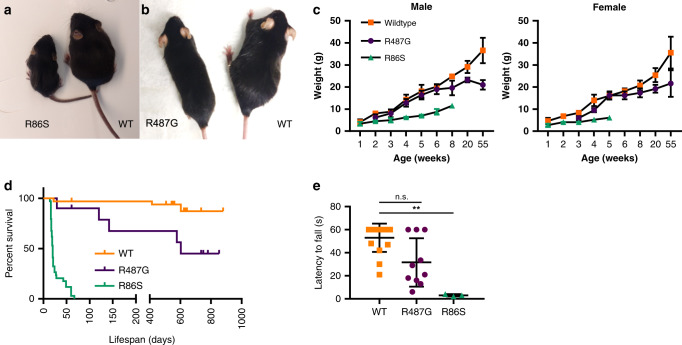
Mutations in mouse *Nemf* lead to deficits in body weight, motor function, and lifespan. **a**, **b** A 18-day-old R86S homozygous mutant (R86S; **a**) and 55-week-old R487G homozygous mutant (R487G; **b**) mice are smaller compared to wild-type (WT) littermates. **c** Body weight curves of male and female wild-type (orange squares), R487G (purple circles), and R86S mice (green triangles). Mean ± SD are indicated for each timepoint. Source data are provided as a source data file. **d** Kaplan–Meier survival curves of males and females combined WT (orange line, *n* = 33), R86S (green line, *n* = 34; median survival: 20 days) and R487G (purple line, *n* = 10; median survival 601 days) mice. Statistical analysis was performed by Log-rank (Mantel–Cox) test with two-tailed *p* value reported R86S vs. WT: *p* ≤ 0.0001 and R487G vs. WT: *p* = 0.0074. **e** Motor function of 8-week-old WT (orange squares), R487G (purple circles), and R86S mice (green triangles) (*n* = 16, 10, and 3, respectively) was measured by wire hang assay. Individual data points and mean ± SD are indicated. Statistical analysis was performed by Kruskal–Wallis test followed by Dunn’s multiple comparisons, R487G vs. WT: *p* = 0.0712 (n.s.), R86S vs. WT: *p* = 0.0018(**), R86S vs. R487G: *p* = 0.1748. Source data are provided as a source data file. For growth curve (**c**) *n* per genotype per time-point: Male: WT (*n* = 5, 6, 7, 6, 5 for weeks 1–6, 7, 8, 20, 55), R86S (*n* = 5, 4, 3 for weeks 1–2, 3–6, 7–8), R487G (*n* = 4, 6, 5, 3, 4, 5, 4 for weeks 2, 3, 4–6, 7, 8, 20, 55). Female: WT (*n* = 4, 3, 5, 4 3,4, 5 for weeks 1, 2, 3, 4, 5, 6, 8–55), R86S (*n* = 3, 5, 1 for weeks 1, 2, 3–5), R487G (*n* = 2, 5, 3, 4, 5, 4 for weeks 2, 3, 4, 5, 6–20, 55).

Through independent ENU mutagenesis screens, two mouse lines with abnormal gait and muscle wasting were identified as carrying homozygous mutations in *Nemf*: C57BL/6J-*Nemf* ^R86S/R86S^ (nucleotide: A258T; protein: R86S) and C57BL/6J-*Nemf*^R487G/R487G^ (nucleotide: A1460G; protein: R487G) (Fig. 1a, b). Henceforth, we refer to these homozygous mutant mice as R86S and R487G, respectively.

R86S mice appear normal at birth but by 2 weeks of age exhibit an overt motor phenotype that manifests as an abnormal waddle-like gait (Supplementary Movie 1), as well as smaller body size (Fig. 1a) with decreased growth rate thereafter (Fig. 1c). In comparison, R487G mice have less marked motor defect and growth reduction (Fig. 1b, c) and only by 10 weeks of age begin to display abnormal gait and hindlimb wasting (Supplementary Movie 2). Disease is progressive in both models. R86S mice die prematurely with a median lifespan of 20 days, with 20% of the animals living past 40 days, whereas R487G mice live past 2 years (Fig. 1d). Both +/R487G and +/R86S (heterozygous animals) have survival and growth rates similar to wild-type mice (Supplementary Fig. 1a, b).

Since *Nemf* mutant mice exhibit abnormal gait, we further examined their motor function. Although the latency to fall in an inverted wire hang assay was comparable for wild-type and R86S mice at 2 weeks of age (Supplementary Fig. 2a), by 8 weeks surviving R86S mice were completely unable to perform the test (Fig. 1e). R487G mice also displayed shorter latency to fall compared to wild-type mice at 8 weeks (prior to overt phenotype onset) but did not exhibit further progression of the defect when examined at 55 weeks of age (Fig. 1e; Supplementary Fig. 2c). Heterozygous +/R86S and +/R487G animals appeared unaffected at all observed timepoints (Supplementary Fig. 2a–c).

### *Nemf* R86S and R487G mice exhibit neurogenic atrophy

To determine the cause of impaired motor function in *Nemf* mutant mice, a histopathological examination of hindlimb muscle was performed. Tissue cross-sections revealed decreased muscle fiber size in the medial gastrocnemius (MG) muscle of 16- to 18-day-old R86S mice compared to wild-type animals (Supplementary Fig. 3a, b). Whereas the MG muscle of R487G mice appeared histologically normal at 2 weeks of age (not shown), by 55 weeks of age localized, nonuniform clusters of atrophied fibers, collagenous fibrosis, and fatty infiltration were observed (Supplementary Fig. 3c).

**Fig. 2:**
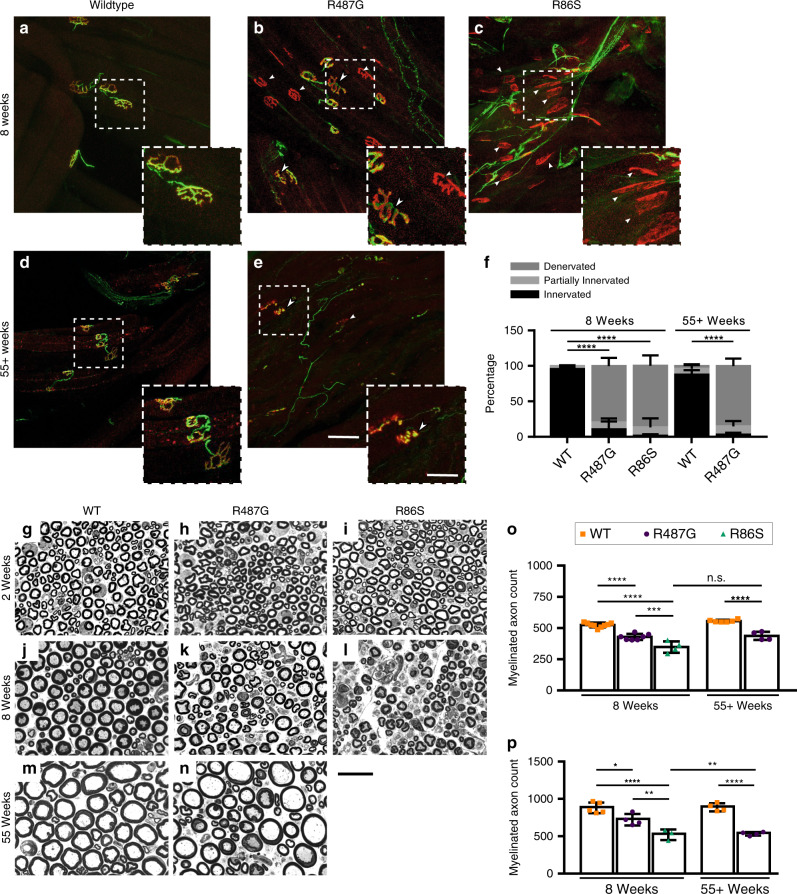
*Nemf* ENU mutations cause progressive neuromuscular degeneration. **a**–**e** Representative images of neuromuscular junction (NMJ) staining of medial gastrocnemius (MG) muscle from mice of indicated genotypes and ages. Tissues were stained for presynaptic vesicles (anti-SV2, green), neurofilaments (anti-neurofilament 2H3, green), and acetylcholine receptor (α-bungarotoxin, red). Unoccupied (arrow) and partially occupied (arrowhead) junctions are highlighted. Scale bar, 60 µm. Inset scale bar, 30 µm. **f** Quantification of fully innervated, partially innervated or denervated NMJs based on presynaptic and postsynaptic staining overlap. Eight-week-old WT, R487G and R86S mice (*n* = 4, 5, and 3 respectively), and 55-week-old WT and R487G mice (*n* = 4 and 3, respectively) were analyzed. By 8 weeks R487G or R86S mice compared to WT (****p < 0.0001), but were not different from one another (*p* = 0.4391). Fifty-five weeks of age, R487G vs. WT (****p < 0.0001). Data are presented as mean percent occupancy value ± SD. Statistical analysis was performed by two-way ANOVA followed by Tukey’s multiple comparison, for % fully occupied. **g**–**n** Representative cross-sections of femoral nerve motor branches from mice of indicated genotypes and ages. Scale bar, 20 µm. **o** Total myelinated axon number in cross-section of WT and *Nemf* mutant mice at 8-week-old wild-type (WT), R487G and R86S mice (*n* = 9, 7, and 4, respectively), and 55-week-old WT and R487G mice (*n* = 6 and 4, respectively). Eight weeks (WT vs. R487G ****p < 0.0001, WT vs. R86S ****p < 0.0001 and R487G vs. R86S ***p = 0.0005). Fifty-five weeks (WT vs. R487G ****p < 0.0001. R487G 8 weeks vs. 55 weeks ^n.s.^*p* = 0.9767). **p** Myelinated axon number in cross-sections of L4 ventral roots of WT and *Nemf* ENU mice. Analysis of 8-week-old WT, R487G and R86S mice (*n* = 5, 3, and 4, respectively), and 55-week-old WT and R487G mice (*n* = 4 each). At 8 weeks of age R487G vs. WT *p = 0.0136, R86S vs. WT ****p < 0.0001, R487G vs. R86S **p = 0.0063, by 55 weeks of age R487G vs. WT ****p < 0.0001, R487G 8 weeks vs. R487G 55 weeks **p = 0.0055. **o**, **p** Individual data points and mean ± SD are indicated. Statistical analysis was performed by one-way ANOVA followed by Tukey’s multiple comparison (wild-type, orange squares; R487G, purple circles; R86S, green triangles). Source data for **f**, **o**, **p** are provided as a source data file.

To investigate the basis of the muscle atrophy, the integrity of neuromuscular junctions (NMJ) was analyzed (Fig. 2a–f), revealing denervation and fragmentation of postsynaptic terminals in 8-week-old R86S mice (Fig. 2a, c, f). At the same timepoint, R487G exhibited more fully and partially occupied junctions than R86S mice (Fig. 2b, f), correlating with the severity of their respective motor phenotypes. Nonetheless, NMJ occupancy was already decreased in R487G compared to wild-type mice at 8 weeks and denervation continued to progress with age (Fig. 2d–f).

### Mutant mice exhibit progressive axonal degeneration

The loss of NMJs suggested that muscle atrophy of *Nemf* mutant mice had a neurogenic origin. To investigate this possibility, we assessed the extent of neuronal degeneration in *Nemf* mutants through the analysis of myelinated axons in the femoral nerve motor branch. At 2 weeks of age, the number of axons were comparable in wild-type and *Nemf* mice (Supplementary Fig. 4a) with similar overall morphology and axonal diameter distribution suggesting that our findings do not reflect a developmental defect (Fig. 2g–i, Supplementary Fig. 4b, c). However, by 16–18 days, R86S mice began to exhibit femoral motor myelinated axon loss (Supplementary Fig. 4a, b), which by late end-stage (8 weeks of age) was further reduced (~30%) (Fig. 2j, l, o) with a shift in axon diameter distribution indicative of the largest fibers being most impacted (Supplementary Fig. 4c). R487G mice also displayed a reduction of the largest diameter fibers at 8 weeks of age (Fig. 2k, o, Supplementary Fig. 4c). Although no further age-related degeneration was observed in R487G animals (Fig. 2m–o), an age-dependent shift in axon diameter distribution towards smaller fibers was observed (Supplementary Fig. 4c). Heterozygous mice exhibited no axonal degeneration, even past 2 years of age; thus, both alleles appear to behave in a purely recessive manner (Supplementary Fig. 4d).

In contrast to peripheral neuropathies, characterized by loss of distal axons such as those in the femoral motor nerve, motor neuron disease can be evidenced by the loss of neuronal cell bodies and their proximal axons, such as the lumbar ventral root exiting from the spinal cord. To determine if mutations in *Nemf* cause a purely peripheral neuropathy or also degeneration of spinal motor axons, lumbar ventral roots (at the L4 level) were examined for cell body-proximal axon loss. As with the distal part of axons, both the R86S and R487G mice exhibited loss of proximal motor axons as early as 16–18 days (Fig. 2p, Supplementary Fig. 5a, b). However, R487G also exhibited further reduction of proximal axons between 8 and 55 weeks of age (Fig. 2p, Supplementary Fig. 5c, d), while distal axons reached terminal degeneration by 8 weeks (Fig. 2o). These results suggest a neuronal dying-back mechanism, wherein the disease originates distally. Consistent with a recessive phenotype, proximal L4 ventral roots also show no difference in heterozygous animals (Supplementary Fig. 5e).

Finally, we note that, although *Nemf* mutant mice exhibit axonal loss associated with motor phenotypes, the disease is not specific to the motor system as preliminary analyses of femoral sensory axons likewise showed a reduction in R86S (but not R487G) animals (Supplementary Fig. 6). Thus, the more severe R86S allele may also cause a sensorimotor neuropathy, similar to *Ltn1* mutant mice^15^.

### NEMF-null mice exhibit an early onset neurologic phenotype

**Fig. 3:**
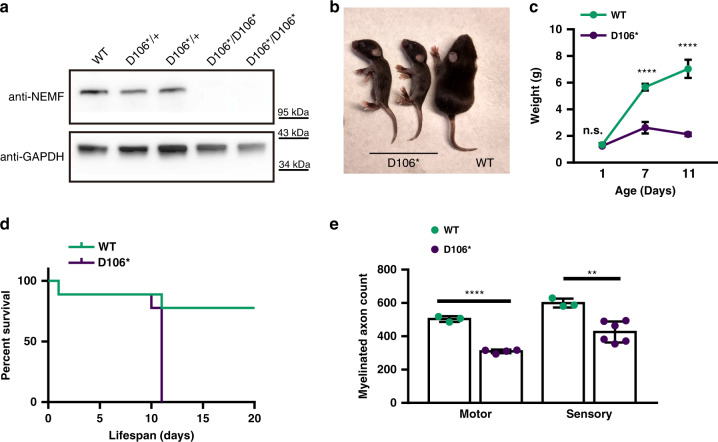
*Nemf* D106* mice display perinatal neurodegeneration and lethality. **a** Western blot analysis of brain lysates from 10-day-old wild-type (WT), and *Nemf* D106* hetero- and homozygous mice using anti-NEMF antibody, and anti-GAPDH antibody as loading control. **b** Eleven-day-old D106* homozygous mice are smaller than WT littermates. Mutant mice display wasting and are unable to upright themselves at this timepoint. **c** Body weight of WT (green circle, *n* = 8 day 1, *n* = 5 days 7 and 11) and D106* homozygous (purple circle, *n* = 4 days 1 and 7, *n* = 7 day 11) mice for WT vs. D106* at each 1, 7, and 11 days of age, respectively (n.s.*p* > 0.9999, ****p < 0.0001, ****p < 0.0001). Mean ± SD is indicated. Statistical analysis was performed by two-way ANOVA followed by Sidak’s multiple comparison. **d** D106* mice have shortened lifespan. Kaplan–Meier survival curves of D106* homozygous (purple line, *n* = 9; median survival: 11 days) and WT (green line, *n* = 9) mice *p* = 0.0022. Statistical analysis was performed by Log-rank (Mantel–Cox) test with two-tailed *p* value reported (**e**), myelinated axon numbers from whole cross-sections of femoral nerve motor branches in 9- to 11-day-old WT and D106* homozygous mice. For motor (WT: *n* = 3; *Nemf* D106*: *n* = 4, ****p < 0.0001) and sensory (WT: *n* = 3; *Nemf* D106*: *n* = 6, **p = 0.0031). Individual data points and mean ± SD are indicated. Statistical analysis was performed by a two-sided unpaired *t* test. Source data for **c**, **e** are provided as a source data file.

Immunoblot analysis of brain and spinal cord extracts indicated that the NEMF ENU mutations did not markedly affect the steady-state levels of the mutant proteins compared to that of the wild-type (Supplementary Fig. 7). In order to assess whether the mutant proteins retain some function (i.e., whether they are hypomorphic), we analyzed mice harboring a putative NEMF-null allele. This allele, generated in the course of CRISPR genetic modifications, has a single nucleotide insertion causing a frameshift and premature termination (nucleotide: 296_297insT; protein: D100G*fs*X6, henceforth, D106*; Supplementary Fig. 8a). Accordingly, no full-length NEMF protein was detected in D106* mouse tissues (Fig. 3a). D106* exhibited more severe phenotypes than R86S or R487G mice, with an early deviation in growth and pre-wean lethality by postnatal day 11 (Fig. 3b–d). Like R86S and R487G, heterozygous D106* mice appear unaffected, consistent with a recessive loss-of-function mechanism (Supplementary Fig. 8b and data not shown). D106* mice exhibited a marked reduction in occupied NMJs in the MG at end-of-life (Supplementary Fig. 8c–e) along with a reduction in peripheral myelinated axons (Fig. 3e, Supplementary Fig. 8f–i). In addition, D106* mice display a respiratory distress phenotype at end-of-life, evident by decreased oxygen saturation (Supplementary Fig. 8j) and phrenic nerve degeneration (Supplementary Fig. 8k–m). Thus, D106* exhibited earlier and more severe neuromuscular changes than R86S and R487G animals. Together, the above results suggest that, similar to the originally described *Ltn1* ENU mutation^15^, the missense *Nemf* ENU mutations are hypomorphic and cause an age-dependent neurodegenerative phenotype, whereas the complete loss-of-function of either *Ltn1* or *Nemf* results in earlier and more severe phenotypes^15^.

### Mouse ENU mutations perturb CATylation activity of NEMF/Rqc2

**Fig. 4:**
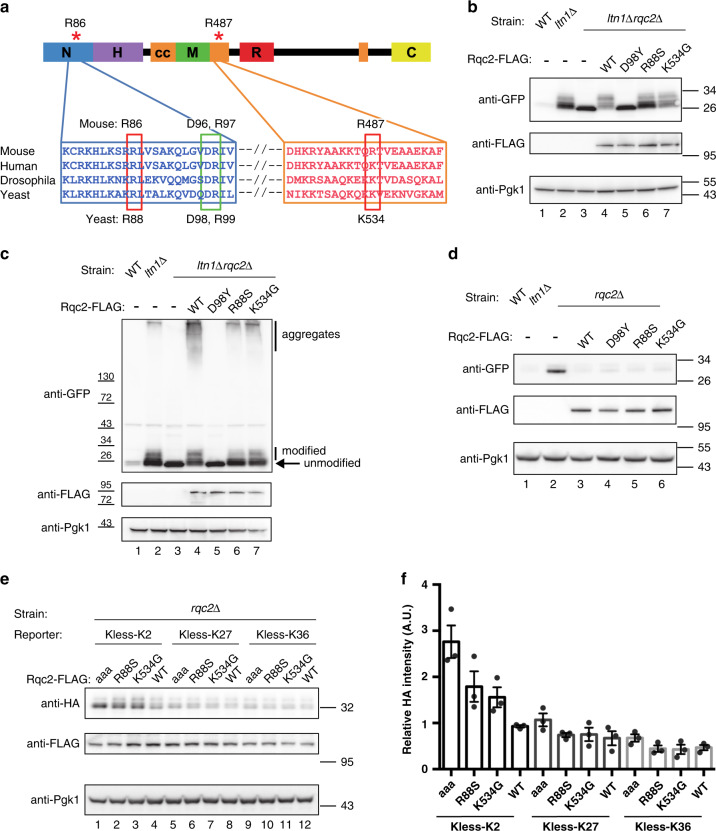
*Nemf* ENU mutations selectively affect the CATylation function of Rqc2. **a** Domain diagram and partial alignment of mouse NEMF and homologs—human NEMF, fly Caliban, and yeast Rqc2. Sequences flanking residues affected by the ENU mutations are shown. Domains and motifs depicted: NFACT-N (N), helix–hairpin–helix (H), coiled-coil (cc), middle domain (M), NFACT-R (R), and NFACT-C (C). NEMF Arg86 and Arg487 mutated in *Nemf* ENU mice (* and red rectangles), and yeast Rqc2 Asp98 and Arg99 residues previously implicated in CAT tail synthesis (green rectangle) are indicated. **b** WT, *ltn1Δ*, or *ltn1Δ rqc2Δ* cells were transformed with the GFP-R12-RFP (GRR) stalling reporter and plasmids encoding Rqc2-FLAG WT, CATylation-deficient D98Y mutant, or ENU-equivalent mutants (R88S and K534G). Immunoblots: anti-GFP and anti-FLAG to monitor reporter and Rqc2 levels, respectively, and anti-Pgk1 as loading control. **c** Strains transformed with Rqc2-FLAG and the GRR stalling reporter, as indicated. Immunoblot anti-GFP monitored reporter modification and aggregation. **d** Wild-type (WT) and *RQC2*-deleted (*rqc2Δ*) cells were transformed with the GRR stalling reporter, and indicated Rqc2-FLAG constructs and analyzed as in “**b**”. **e** Kless-K2, -K27, or -K36 reporters (see Supplementary Fig. 8) expressed in *rqc2Δ* cells together with Rqc2-FLAG WT, the CATylation-deficient Rqc2 aaa mutant (Asp9, Asp98, and Arg99 mutated to Ala), or Rqc2-equivalent ENU mutants as indicated. Anti-HA and anti-FLAG blots monitored reporter and Rqc2 expression, respectively. **f** As in panel “**f**”. Quantification of reporter levels relative to the Pgk1 internal control from three biological replicates, individual values with means ± SEM. For each blot **b**–**e** MW markers in kDa are indicated.

The observation that NEMF ENU mutant proteins are expressed at normal levels implied a defective function, so we next investigated the molecular consequences of those mutations. Notably, the R86S mutation maps to the NFACT-N domain—which has been shown to mediate C-terminal tailing in organisms as divergent as yeast and bacteria^11,13^. The R86S mutation is in the vicinity of the universally conserved, catalytic residues D96 and R97 (Fig. 4a). With regard to the R487G mutation, it maps to the structurally adjacent second coil motif, which is also likely to contribute to C-terminal tailing by assisting with A-site tRNA recruitment^11^.

Since NEMF homologs are well conserved with regard to sequence, structure and function and robust assays for RQC activity are established in yeast, we utilized this model organism for mechanistic studies. Although mammalian NEMF could not functionally replace Rqc2 in yeast (unpublished observations), the residues affected by the ENU mutations in mice are also conserved in yeast (Fig. 4a, Supplementary Fig. 9) so the equivalent mutations were introduced in Rqc2 (R88S and K534G, respectively; Fig. 4a). The equivalent residue of NEMF’s universally conserved D96 in yeast is D98 (Fig. 4a), which was mutated to Tyr^16^. Rqc2 R88S, K534G, and D98Y mutant levels were comparable to the wild-type protein (Fig. 4b–f).

To investigate the effect of the ENU mutations in C-terminal tailing by Rqc2, we used Ltn1-deficient yeast, since, as previously reported, in *ltn1*Δ cells a ribosome stalling reporter became readily modified with CAT tails (as evidenced by the formation of a smeared band immediately above the unmodified reporter band; Fig. 4b, c, lanes 2)^11,16,17^. As also reported, CATylation was strictly dependent on Rqc2^11,16–18^: this modification was abolished in Ltn1- and Rqc2-double deficient cells (Fig. 4b, c, lanes 3) and could be restored by expression of Rqc2 wild-type (lanes 4), but not by a rationally designed, CATylation-deficient D98Y mutant (lanes 5)^16^. As predicted by the hypothesis, Rqc2 ENU mutants were also defective in CATylation in this assay, although only partially (lanes 6–7). Furthermore, as indicated by the ratio of modified versus unmodified species, the Rqc2 R88S substitution caused a stronger defect in CAT tail formation in comparison to the K534G; this finding shows a striking correlation with the phenotypic severity of the respective mouse alleles (Fig. 4b, c, lanes 6–7).

Importantly, additional functions of Rqc2 in RQC remained unaffected by the ENU mutations. As previously reported, levels of a ribosome stalling reporter were low in wild-type yeast due to Ltn1-mediated degradation^6^ (Fig. 4d, lane 1 (anti-GFP)) and were increased in cells lacking Rqc2 (*rqc2*Δ; lane 2) likely due to less efficient Ltn1 binding^7,8,11,14,16–18^. Re-expression of wild-type Rqc2 (lane 3), or the “ENU mutant” Rqc2 proteins (lanes 5 and 6) rescued this *rqc2*Δ phenotype in an otherwise wild-type background, indicating that the mutant proteins are competent to bind to ribosomes and stabilize Ltn1 binding. Thus the ENU mutations appear to cause a selective defect in C-terminal tailing.

To provide further evidence for the CATylation defect of Rqc2 ENU mutants, we analyzed expected consequences of impaired CATylation. For example, CAT tails are known to promote nascent chain aggregation when Ltn1-mediated ubiquitylation fails altogether^16–18^. Accordingly, in *ltn1*Δ cells (or in *ltn1*Δ *rqc2*Δ cells expressing wild-type Rqc2), CAT tail-mediated aggregation of a ribosome stalling reporter was conspicuous (Fig. 4c). In this assay, both Rqc2 ENU mutants were likewise competent to mediate reporter aggregation in *ltn1*Δ *rqc2*Δ cells; however, consistent with these mutants’ decreased ability to make CAT tails, aggregates were formed to a lower extent compared to wild-type Rqc2. Similar observations were made when the formation of endogenous aggregates were monitored by using as a readout, the hsp40 Sis1, which becomes stably incorporated into CAT tail-dependent aggregates^16–18^ (Supplementary Fig. 10a).

CATylation has been additionally implicated in facilitating Ltn1-mediated ubiquitylation^14^. As expected, such a requirement was not evident in the results of the experiment shown in Fig. 4d, since the ribosome stalling reporter utilized in that experiment was rich in Lys residues and was predicted to present Lys for Ltn1-mediated ubiquitylation even in the absence of added CAT tails^14^. We thus engineered an alternative reporter whose Ltn1-mediated ubiquitylation and degradation would be dependent on CAT tail synthesis, because the reporter lacked Lys residues (K-less) except at two positions buried deep within the ribosomal exit tunnel, located 2 residues N-terminal to the ribosome stalling sequence (Kless-K2; Supplementary Fig. 10b). Consistent with a CAT tail requirement for degradation, Kless-K2 reporter levels (anti-HA) were indeed higher in *rqc2*Δ cells reconstituted with a CATylation-deficient Rqc2 mutant (Rqc2 aaa^11^) compared to Rqc2 wild-type (Fig. 4e, f). Notably, Kless-K2 levels were intermediate in cells expressing the Rqc2 ENU mutants consistent with those mutants’ partial CATylation defect. As controls, reporters with Lys residues either closer to the opening of the exit tunnel (Kless-K27) or predicted to be already exposed on the outside (Kless-K36) were also tested. As expected, the levels of these reporters in the presence of Rqc2 ENU mutants were low and comparable to the Rqc2 wild-type and aaa mutant (Fig. 4e, f). We additionally verified that all constructs were targeted for degradation in a Ltn1-dependent manner (Supplementary Fig. 10c). These results substantiate the model that CATylation can function to support Ltn1-mediated ubiquitylation and suggest that the Rqc2 ENU mutations interfere with the degradation of a subset of Ltn1 substrates that rely on CAT tails for ubiquitylation.

### *NEMF* variants are associated with juvenile neuromuscular disease

Through collaboration facilitated by GeneMatcher^19^, nine patients from seven unrelated families with likely pathogenic variants in *NEMF* were identified. Seven patients harbored biallelic variants, an eighth patient harbored an inherited variant and a de novo variant (though the chromosomal phase is undetermined, i.e., whether the variants are in *cis* or *trans*) and the ninth patient harbored a single de novo missense variant. These patients display intellectual disability and/or early motor neuron disease phenotypes of varying severities and progression, summarized in Table 1. Detailed clinical findings are available (Supplementary Note 1).

**Table 1:** Clinical features of affected individuals.

Family	AUS1	DEU1	DEU1	IRN1	SAU1	SAU1	USA1	USA2	USA3
Individual	II:3	II:3	II:6	II:1	II:1	II:2	II:1	II:1	II:2
Age (years)	7 + 7 mo	22	16	28	10 + 6 mo	7	6 + 4 mo	7 + 10 mo	24
Sex	M	M	F	M	M	M	F	F	M
Age at onset (years)	1.5	2	1	1	2	2	1.7	2	17
ID/GDD	+	+	+	+	+	+	−	+	−
Speech delay	+	+	+	+	+	+	+	+	+
Neuropathy (axonal)	+	+	+	N/A	N/A	N/A	N/A	N/A	+
Ataxia	+	+	+	−	−	−	−	−	−
Distal muscle atrophy	+	+	+	+	−	−	−	−	+
Hypotonia	−	−	+	+	−	−	+	+	−
Respiratory distress	−	−	−	−	−	−	+	−	−
Tremor	+	−	−	N/A	−	−	−	−	+
Abnormal brain imaging	N/A	+	−	N/A	−	−	N/A	+	N/A
Kyphosis/Scoliosis	+	+	+	−	−	−	+	−	−

*ID/GDD* intellectual disability/global developmental delay; − absent, + present, *N/A* not ascertained/not applicable.

**Fig. 5:**
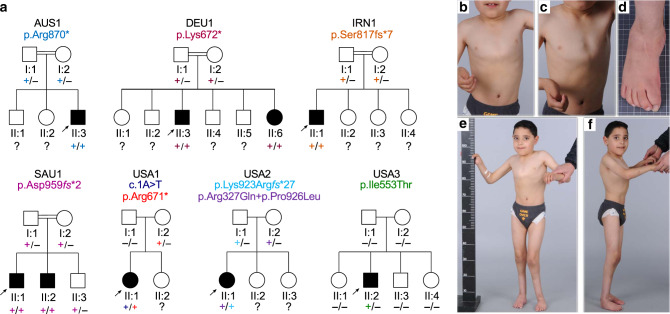
NEMF variants are associated with neuromuscular disease in humans. **a** Pedigrees of identified families having affected individuals and NEMF variants. “−” indicates wild-type allele, “+” indicates variant allele, arrowhead indicates proband. Different font colors are used to facilitate visualization of the distribution of wild-type (“−”) and unique mutant (“+”) alleles in the pedigrees. **b**–**f** Clinical photography of 7-year-old subject AUS1-II:3. Asymmetric pectus excavatum, scoliosis (**b**), and characteristic adducted thumb posture are evident (**c**). Right foot showing surgical scars with residual equinovarus foot deformity and toe clawing (**d**). Images showing pectus excavatum, kyphoscoliosis, hip, and knee flexor contractures with distal muscle wasting and residual equinovarus foot posturing despite previous orthopedic surgery (**e**, **f**).

An Australian case of consanguineous Syrian parents (AUS1-II:3, Fig. 5a–f) presented with axonal neuropathy and spastic paraplegia at 18 months of age; the individual has severe speech delay. Whole-genome sequencing (WGS) identified a homozygous nonsense variant in *NEMF* (c.2608C > T, p.(Arg870*)). In a Turkish family, (DEU1, Fig. 5a) with two individuals presenting with developmental delay and peripheral neuropathy in childhood, a homozygous nonsense variant in *NEMF* was identified (c.2014A > T, p.(Lys672*)). In a Persian family (IRN1, Fig. 5a), a proband presented with hypotonia, developmental delays and severe speech delay; WGS identified a homozygous frameshift variant in *NEMF* (c.2451delA; p.(Ser817fs*7)). In a Saudi Arabian family (SAU1, Fig. 5a) with two individuals presenting with developmental delays, a homozygous frameshift variant in *NEMF* was identified (c.2871_2875dupTGTAG; p.(Asp959fs*2)). In a Caucasian proband (USA1-II:1, Fig. 5a) presenting with gross developmental delay in the second year of life, two *NEMF* variants were identified: a maternally inherited nonsense variant (c.2011C > T; p.(Arg671*)) and a de novo variant of the start codon (c.1A > T; p.(Met1?)). Chromosomal phase could not be determined based on clinical NGS sequencing. In another Caucasian proband (USA2-II:1, Fig. 5a), who presented with mild hypotonia and speech delays, biallelic *NEMF* variants were identified by exome sequencing: two maternally inherited missense variants (c.980G > A;p.Arg327Gln and c.2777C > T; p.Pro926Leu) and a paternally inherited frameshift variant (c.2768delA; p.Lys923Argfs*27). A Caucasian proband (USA3-II:2, Fig. 5a) who presented with axonal sensorimotor peripheral neuropathy at 17 years of age harbored a de novo missense variant in *NEMF* (c.1658T > C, p.(Ile553Thr)). The variant is absent from gnomAD and predicted to be damaging by all protein prediction programs utilized (CADD “score 28”; MutationTaster, “disease causing”; PolyPhen-2, “probably damaging”; Provean, “deleterious”; SIFT, “deleterious”). The substituted p.(Ile553) is conserved through evolution including in yeast (Supplementary Fig. 9). As a second disease-associated variant has yet to be identified, it is possible that the de novo p.(Ile553Thr) variant acts alone in a dominant-negative manner.

Thus, we present eight cases from six families with biallelic, likely pathogenic or pathogenic variants (based on American College of Medical Genetics (ACMG) guidelines^20^), and a ninth case with a milder phenotype carrying a de novo missense variant affecting a highly conserved residue. These findings suggest that variants in *NEMF* can cause neuropathy and motor dysfunction, sometimes associated with speech delay and intellectual disability.

## Discussion

Our studies identify *Nemf* as a novel gene implicated in neurodegeneration and neuromuscular disease in mice and humans. Moreover, this finding, along with the previously described *Ltn1* mouse model^15^, strongly point to RQC as a critical molecular pathway protecting neurons against degeneration. This suggests that the presence of variants in *NEMF* and other RQC factors (e.g., Listerin) should be examined in patients with related diseases.

Our results indicate that patients with *NEMF* variants can additionally manifest intellectual disability, most notably speech delay. This conclusion is supported by a genome-wide association analysis which included *NEMF* variants among several genes potentially implicated in cognitive phenotypes^21^. Similarly, mutation of *GTPBP2*, a GTPase that mediates the splitting of stalled ribosomes upstream of RQC^22^, causes Jaberi–Elahi syndrome^23–25^, an early onset neurodegenerative disease characterized by dystonia, motor and sensory neuropathy, ataxia, and cognitive dysfunction. Future experiments will address the potential for cognitive dysfunction in *Nemf* mutant mice, although we anticipate that the interpretation of such analyses may be confounded by the presence of motor and sensory deficits.

The observation that one patient (USA3-II:2) with a heterozygous variant and later onset peripheral neuropathy phenotype may suggest potential dominant-negative impacts from variants in *NEMF*. Future work will address potential modifiers and determine if this allele could dominantly cause disease in mouse models. Thus far, no motor or sensory peripheral degeneration has been observed in our heterozygous mouse models at one and a half years of age.

The observations that Rqc2 homologs from bacteria to humans have high overall sequence and structural similarity and that residues specifically implicated in the C-terminal tailing modification are strictly conserved (Fig. 4a, Supplementary Fig. 9; refs. ^2,11,13^) strongly suggests that Rqc2 homologs across all domains of life share a fundamental role in mediating C-terminal tailing in RQC. In fact, this biochemical activity has been reported for a homolog as evolutionarily distant as *B. subtilis* RqcH^11,13^. Despite great progress made toward understanding this and other fundamental mechanisms in RQC^2^, new mammalian models have been lacking to establish the physiological relevance of the findings and to directly test predictions regarding molecular mechanisms of neurodegeneration. Our results now show that the neurodegeneration-causing mouse NEMF R86S and R487G mutations selectively interfered with yeast Rqc2’s ability to mediate CAT tail synthesis, and that phenotypic severity is correlated with the extent of their reduction in CATylation activity, thus suggesting both a link between neurodegeneration and defective C-terminal tailing as well as a physiological role for this RQC mechanism in mammals.

## Methods

### ENU Mutagenesis and mapping

The B6J-*Nemf* ^R86S^ line was originally derived from an *N*-ethyl-*N-*nitrosourea mutagenesis screen for reproductive defects initiated at The Jackson Laboratory^26^. Chemical mutagenesis was induced in a C57BL/6J background and mutagenized mice were subsequently outcrossed to C3HeB/FeJ (C3H). The line was then backcrossed to C57BL/6J for at least 10 generations to obtain the B6J-*Nemf* ^R86S^ (JAX Stock #19392) line used in this study. The mutation was mapped to a 4.6 Mb interval in an F2 intercross (Chr. 12: 65486719-70085250 bp, GRCm38) and the only exonic variant detected among 15 candidate genes analyzed was in the *Nemf* gene. The B6J-*Nemf* ^R487G^ line (JAX Stock #18870) was derived from an independent ENU mutagenesis screen for eye phenotypes in a C57BL/6J background, also at The Jackson Laboratory^27^. Crossing heterozygous animals from each ENU allele produced affected offspring in an allelism test suggesting the two mutations were in the same gene. Both strains were mapped to Chr. 12 in F2 intercrosses^26,27^ and each allele was backcrossed to C57BL/6J for at least ten generations to eliminate unlinked and distantly linked ENU-induced mutations as potential genetic confounds. A positive allelism test between *Nemf* ^R86S^ and *Nemf* ^R487G^ confirmed that the ENU-induced mutations were disease-causing and were not the result of an unknown, but tightly-linked alternate ENU mutation.

### CRISPR–*Cas9* mutagenesis

The *Nemf*^D106*^ strain (JAX Stock #34815) was obtained from a CRISPR–Cas9 mutagenesis performed by cytoplasmic microinjection of C57BL/6J zygotes with *Cas9* mRNA and sgRNA targeting exon 4 of *Nemf*: 5′-GCTTGGTGTGGACAGAATTG-3′. Mosaic founder mice identified as carrying a mutation in the targeted region were backcrossed to C57BL/6J. Resulting N1 progeny identified as carrying the mutation were further backcrossed to C57BL/6J to establish the colony.

### Mouse strains, husbandry, and genotyping

All mouse husbandry and procedures were reviewed and approved by the Institutional Animal Care and Use Committee at The Jackson Laboratory were carried out according to the NIH Guide for Care and Use of Laboratory Animals (AUS# 01006). Mice were bred and maintained under standard conditions: an ambient room temperature of 69 °F, humidity at 42% and a 10/14 h dark/light cycle. Tail or ear tissue was lysed in proteinase K at 55 °C overnight and extracted DNA was used to determine genotype. At The Jackson Laboratory, genotyping for B6J-*Nemf* ^R86S^ strain was performed via PCR using the following primers: forward primer specific to wild-type allele: 5′-AACATTTGAAGAGTCGGGGA-3′; forward primer specific to mutant allele: 5′-AACATTTGAAGAGTCGGGGT-3′; reverse primer common for both alleles: 5′-GCAGGTGGATGGTAGCAACG-3′. Similarly, for the genotyping of the B6J-*Nemf* ^R487G^ mice the following primers were used: forward primer specific to wild-type allele: 5′-TGCTGCTAAAAAAACCCGGA-3′; forward primer specific to mutant allele: 5′-TGCTGCTAAAAAAACCCGGG-3′; reverse primer common for both alleles: 5′-AAAGCCCTTGCTGCAAAGCC-3′. The following primers were used for the genotyping of B6J-*Nemf* ^D106*^ strain: forward primer: 5′-CATGGTGAATGGAGAGAACC-3′; reverse primer: 5′-TTGATCCCAGCACTAGGGAG-3′. D106* PCR product was Sanger sequenced and assessed via chromatogram.

### Phenotypic and behavioral analyses

Mice were weighed weekly from 7 ± 1 days of age to 8 weeks, unless indicated otherwise. Longer surviving animals were additionally weighed at 20 and 55 weeks. For the D106* mouse weight at weeks 1 and 1.5, it was determined that there were no sex differences on the phenotypes and therefore the data was presented as mixed sex, with similar numbers of males and females. Inverted wire hang assay was used to assess motor function. It was determined that there were no sex differences in phenotype and therefore the data was presented as mixed sex, with similar numbers of males and females: mice were placed on top of a wire mesh cage cover which was then inverted for a maximum of 60 s, and the latency to fall was measured. Mice were allowed to rest for a minimum for 5 min before repeating the test. The average of two tests was recorded per timepoint.

### Analysis of myelinated axons

Motor and sensory branches of the femoral nerve, L4 ventral roots or phrenic nerves were dissected and fixed overnight at 4 °C in 2% glutaraldehyde and 2% paraformaldehyde in 0.1 M cacodylate buffer. Nerves were then post-fixed in 1% osmium tetroxide in 0.1 M cacodylate buffer and embedded in Embed 812 Resin (Electron Microscopy Sciences, Hatfield, PA). Sections measuring 1 µm were cut on a Leica RM2265 rotary microtome with a diamond knife, baked onto a glass slide, and heat stained with 0.5% aqueous toluidine blue. For myelinated axon count and diameter analyses, images were captured using a Nikon Eclipse E600 microscope with 40× and 100× objectives. The total number of myelinated axons in each nerve was counted using an automated threshold method in ImageJ (v1.52p) with manual confirmation^28^. With ImageJ software (v1.52p), the Threshold function was adjusted in order to only highlight axoplasm on whole nerve sections, and Analyze particle function was then used to quantify the number of myelinated axons and areas of each nerve. The diameter was determined from axonal area. It was determined that there were no sex differences in phenotype and therefore the data was presented as mixed sex, with similar numbers of males and females. Images of large nerves that could not be captured as a single image at 40× magnification were generated as montages to show the whole nerve, using the ImageJ (v1.52p) Stitching Grid/Pairwise plugin^29^.

### Histological analysis of gastrocnemius muscle

Mice were euthanized and right hindlimb was extracted and post-fixed in Bouin’s fixative. Whole hindlimbs were cross-sectioned through the middle portion of lower and upper legs. The sectioned tissues were then paraffin-embedded, sectioned, mounted, and stained with hematoxylin and eosin for light microscopic analysis according to standard histological procedures. Slides were scanned using a NanoZoomer 2.0 (Hamamatsu) at 40× magnification. Representative images were taken from the MG region of the lower leg, using NDP.view2 software (U12388-21, Hamamatsu).

### NMJ staining and occupancy analysis

The MG muscle was dissected and fixed in freshly prepared 2% paraformaldehyde (Electron Microscopy Sciences) in phosphate-buffered saline (PBS) overnight. The samples were then incubated in blocking solution (2.5% bovine serum albumin (Sigma-Aldrich) and 1%Triton-X 100 (Sigma-Aldrich) in PBS) for 1 h before they were gently teased apart and pressed between two glass slides using a binder clip for 15 min at 4 °C. Samples were subsequently returned to the blocking solution, permeabilized overnight at 4 °C, and incubated with primary antibodies (1:500 mouse monoclonal IgG_1_ anti-neurofilament 2H3 and 1:250 anti-SV2 (DSHB) in blocking buffer) overnight at 4 °C on a slow shaker. Samples were then washed at least 4 times for 15 min in 1× PBS and incubated overnight at 4 °C on a slow shaker with 1:500 Alexa-Fluor 488 goat anti-mouse IgG_1_ and 1:1000 α-bungarotoxin conjugated with Alexa-Fluor 594 (Invitrogen, Carlsbad, CA) to stain for acetylcholine receptor (AChR). After incubation, samples were rinsed three times and washed at least four times for 15 min each, mounted in 80% glycerol and imaged using an SP5 Leica confocal microscope. Occupancy of 50 or greater randomly selected NMJs was scored blinded to genotype on a Nikon E600 fluorescence microscope. Full occupancy of NMJ was defined as when presynaptic nerve staining fully overlaid with AChR, partial occupancy as when positive for AChR but only partially stained for presynaptic nerve, and denervation as when positive for AChR but negative for presynaptic nerve staining. It was determined that there were no sex differences in phenotype and therefore the data was presented as mixed sex, with similar numbers of males and females. Images were obtained on a Leica SP5 or SP8 laser-scanning confocal microscope with a 40× or 63× objective lens. Z stacks were collapsed into projected images and merged using FIJI (v1.52p)^30^ (NIH, http://rsb.info.nih.goc/ij/).

### Oxygen saturation measurements

The MouseOx^TM^ Pulse-oximeter (Starr Life Sciences, Oakmont PA) was used to measure SpO2 on 10- and 11-day-old D106* mice and unaffected littermates. It was determined that there were no sex differences in phenotype and therefore the data was presented as mixed sex, with similar numbers of males and females. Mice were measured in a darkened room and held until calm, before placing the extra-small collar probe on the neck with the reading window near the carotid artery. Readings were taken for approximately 2–10 min from each mouse. Readings were sorted by Excel spreadsheet and R, for readings that had no SpO2 error codes in a block.

### *S. cerevisiae* strains and constructs

Strains used in this work are isogenic to BY4741^16^. The GFP-Arg12-RFP (referred to as GRR) construct was a gift of J. Weissman (UCSF)^7^. To generate Rqc2 constructs, the coding sequence of *Saccharomyces cerevisiae RQC2* was amplified by PCR with the 3× FLAG epitope added at the C-terminus, and cloned into the YCplac111 vector (*LEU2* marker, *CEN*, and GPD promoter)^16^. Point mutations were inserted by site directed mutagenesis or by gene synthesis. To generate the Rqc2 aaa mutant^11^, a gene fragment was synthesized to contain the first 181 amino acids of Rqc2 with D9A, D98A, and R99A mutations flanked with 5′ PstI and 3′ SpeI restriction sites (IDT). The gene fragment was subcloned into the Rqc2-3× FLAG construct by digestion with SpeI and PstI followed by triple ligation. The Kless-K2, -K27, and -K36 reporters were generated by inverse PCR of the HA-Kless GFP-R12 construct^16^ to introduce additional Lys-Ala-Gly-Lys (AAAGGTGCTAAA) sequences in amino acid positions 2, 27, and 36 N-terminal of the R12 stalling signal.

### Protein expression analyses

Yeast total soluble extracts were prepared from cells that were flash frozen in liquid nitrogen and lysed with glass beads under denaturing conditions. Protein quantitation was performed by the BCA method. Totally, 7.5–30 µg of protein extract were resuspended in sample buffer (1% sodium dodecyl sulfate, 0.005% bromophenol blue, 5% glycerol, 50 mM dithiothreitol, 50 mM Tris-Cl (pH 6.8)) and incubated at 100 °C for 5 min. Protein samples were run on Novex or Novus gels (Invitrogen), transferred onto polyvinylidene fluoride (PVDF) membrane and immunoblotted for anti-FLAG tag (M2, Sigma, 1:1000), anti-HA (3E10, Roche. 1:1000), anti-GFP (Roche clone 7.1 and 13.1, 1:1000), anti-Pgk1 (Invitrogen clone 22C5D8, 1:10,000) and anti-Sis1 (E. Craig^31^, 1:10,000-20,000). Blots corresponding to yeast work (Fig. 4b–e, Supplementary Fig. 10a, c) are available uncropped in Supplementary Figs. 11 and 12.

For mouse experiments, spinal cord and brain were collected immediately following cervical dislocation, snap-frozen in liquid nitrogen and stored at −80 °C. Tissues were homogenized in lysis buffer (20 mM Tris-HCl (pH7.5), 150 mM NaCl, 5 mM EDTA (pH8), protease inhibitor (Complete Protease Inhibitor Cocktail, Roche)) on TissueLyserII (Qiagen), incubated in 0.5% NP-40 for 30 min at 4 °C, and precleared at 12,000 RCF for 20 min. Protein concentrations were determined by BCA method. Protein samples were boiled in sample buffer and were run on ExpressPAGE gels (Gene Script), transferred onto PVDF membrane and immunoblotted with anti-NEMF (Proteintech, 11840-1-AP, 1:1000) and anti-GAPDH (14C10, Cell Signaling,1:20,000) antibodies. Immunoblots were developed using ECL reagent (Thermo Fisher, GE healthcare) and imaged in LAS 4000 Imager or on X-ray film. Uncropped blots from Fig. 3a and Supplementary Fig. 7 are available in Supplementary Figs. 11 and 12.

### Sequence alignment

Amino acid sequences were aligned using CLUSTAL W(V2.1)^32^ and files were generated by Strap software (September 2012 version)^33^. Sequence similarity was calculated using the Sequence Manipulation Suite(V2.0)^34^. Alignment plot was generated by PLALIGIN(V2.0)^35^.

### Ethics approval

The participants in this study were identified through the GeneMatcher database^19^ where clinicians and researchers with variants in genes can identify groups with similar genotype/phenotype findings and collaborate. Informed consent was obtained from all individuals included in the study. Approval for the studies have been from the following: The Institutional Review Board (IRB) at Baylor College of Medicine (protocol number H-29697); considered IRB exempt at the University of South Florida and Mission Fullerton Genetics Center; The Australian Genomics Neuromuscular Disorders ethics committee approval from Melbourne Health (HREC/16/MH251); approval from the ethics commission of Otto-von-Guericke-Universität and the Medizinischen Fakultät at Universitätsklinikum Magdeburg A.ö.R.; the IRB at The University college London; the IRB at King Fahad Medical City (IRB#19-512). The authors affirm that human research participants provided informed consent for the publication of the images in Fig. 5b.

### Patient DNA sequencing

#### USA1 and USA2

Exons and flanking splice junctions were captured from genomic DNA using the IDT xGen Exome Research Panel v1.0. Massively parallel sequencing (NextGen) was performed on an Illumina system with 100 bp or greater paired-end reads. Reads were aligned to human genome build GRCh37/UCSC hg19, and analyzed for sequence variants using a custom-developed analysis tool. Whole-exome sequencing (WES) data for all sequenced family members was analyzed using GeneDx’s XomeAnalyzer. This includes nucleotide and amino acid annotations, population frequencies (NHLBI Exome Variant Server, 1000 Genomes, and internal databases), in silico prediction tools, amino acid conservation scores, and mutation references. The identified variants were filtered based on inheritance patterns, variant type, gene lists of interest developed internally, phenotype, and population frequencies, as appropriate^36^. The general assertion criteria for variant classification are publicly available on the GeneDx ClinVar submission page^37^.

USA3Clinical exome sequencing was carried out at Baylor Genetics Laboratories as part of the clinical work up and his parents’ DNAs were sequenced at the Human Genome Sequencing Center at Baylor College of Medicine as part of the Baylor-Hopkins Center for Mendelian Genomics (BHCMG) research initiative. Briefly, genomic DNA underwent exome capture with Baylor College of Medicine Human Genome Sequencing Center core design (52 Mb, Roche NimbleGen, RRID: nif-0000-31466), and were sequenced on the HiSeq platform (Illumina) with ∼100× depth of coverage. Using the Mercury in-house bioinformatics pipeline, sequence data was aligned and mapped to the human genome reference sequence (hg19)^38^.

#### AUS1

Whole genome sequencing (Illumina HiSeq) of the proband was performed at Kinghorn Center for Clinical Genetics, Garvan Institute of Medical Research. Data were analyzed using the Seave bioinformatic analysis pipeline^39^.

#### DEU1

WES was performed with leukocyte genomic DNA from both affected children using the capture library SureSelect^XT^ Human All Exon v5 + UTRs (Agilent Technologies) for enrichment and sequencing on a Illumina HiSeq2500 instrument (2 × 100 bp, paired end run). The mean coverage of the target sequence was 50×. The obtained sequence data was analyzed using the Varvis^®^ genomics platform v1.12.0 (Limbus Medical Technologies GmbH).

IRN1: WES of proband’s DNA was performed. Exomes were enriched with HiSeq 3000 to an average sequence depth of 91×. Variants were called and annotated with ANNOVAR^40^. In accordance with the recessive mode of inheritance, priority was given to rare biallelic functional variants with allele frequency <0.001% in public databases, including 1000 Genomes project, NHLBI Exome Variant Server, Iranome. GME Varome and gnomAD as well as our in-house database consisting of 14,000 exomes. No plausible compound heterozygous or homozygous variants were identified in genes previously associated with neurological phenotypes.

#### SAU1

Clinical Exome Sequencing (BGI Europe) was carried out using an Illumina HiSeq 4000 to an average depth of coverage of 150× with automated adapter trimming of the fastq sequences. DNA sequence quality metrics were carried out using FASTQC version: 0.11.7. Alignment, quality filtering and variant identification were undertaken using commercially available algorithms. Human reference assemblies were aligned against GRCh37.p13. QIAGEN Clinical Insight—Interpret software was used in sequence analysis and interpretation. Confirmatory Sanger test was performed for the index case as well as parent and two siblings.

### Statistics and reproducibility

Statistical tests were performed using GraphPad’s Prism (v7) software. A threshold of *p* < 0.05 was considered significant. Normality of distribution was determined using the Shapiro–Wilk normality test with GraphPad’s Prism (v7). For assays with data displayed with combined sex, it was determined that there were no sex-dependent differences in phenotype and similar numbers of males and females were used. Outliers were not removed from phenotypic data. Significance was determined using the test indicated in the figure legends. Results are presented as means ± SD, with individual data points where *n* < 10.

For reproducibility, NMJs and axon counts were analyzed genotype blind. For the representative panels Fig. 2a–e are associated with the data collected in Fig. 2f. Representative images in Fig. 2g–n are associated with the data collected in Fig. 2o. Representative image panel Supplementary Fig. 4b is associated with data collected in Supplementary Fig. 4a. Representative image panels Supplementary Fig. 5b–d are associated with the data collected in Fig. 2p and Supplementary Fig. 5a. The representative image panel Supplementary Fig. 4k–l is associated with data collected in Supplementary Fig. 4j. The representative image panels Supplementary Fig. 8c, d are associated with the data collected in Supplementary Fig. 8e. The representative image panel in Supplementary Fig. 8f–I are associated with data collected in Fig. 3e. The representative image panels in Supplementary Fig. 8l, m are associated with data collected in Supplementary Fig. 8k.

Supplementary Fig. 3a, b are representative of *n* = 4 WT and *n* = 4 R86S and Supplementary Fig. 3c is representative of *n* = 8 WT and *n* = 4 R487G biological replicates. For yeast assays (Fig. 4 and Supplementary Fig. 10), once assay conditions were defined, experiments were independently replicated successfully at least 3 times and representative data are shown. For mouse protein assays (Fig. 3a, Supplementary Fig. 7a, b), protein lysates of at least four mice for each strain and genotype and multiple organs were tested to confirm lack of NEMF expression. All attempts were successful.

### Reporting summary

Further information on research design is available in the Nature Research Reporting Summary linked to this article.

## Supplementary information

Supplementary Movie 1

Supplementary Movie 2

Supplementary Information

Peer Review File

Description of Additional Supplementary Files

Reporting Summary

## Data Availability

The data that support the findings of this study are available as source data in the data source file. Variant information will be available upon reasonable request and have been submitted to ClinVar (https://www.ncbi.nlm.nih.gov/clinvar/?term=NEMF%5Bgene%5D)^37^, AnVil (https://anvilproject.org/) and the European Genome-phenome Archive (https://www.ebi.ac.uk/ega/home) for public access. Data for genetic matches with NEMF variants can be found on GeneMatcher^19^ available to clinicians and researchers describing patients with variants of interest. The variant summary statistics work makes use of the following publicly available databases: ClinVar (https://www.ncbi.nlm.nih.gov/clinvar/?term=NEMF%5Bgene%5D)^37^, Genome Aggregation database (Gnomad.V2.1.1, GRCh37/hg19; https://gnomad.broadinstitute.org/)^41^, Greater Middle East (GME) Variome (V1) (http://igm.ucsd.edu/gme/)^42^, Iranome (http://www.iranome.ir/)^43^, and 1000 Genomes Project (https://www.internationalgenome.org/1000-genomes-browsers)^44^. Source data are provided with this paper.

## Source data

Source Data
