# Author Correction: NEMF mutations that impair ribosome-associated quality control are associated with neuromuscular disease

**DOI:** 10.1038/s41467-020-18941-4

**Published:** 2020-10-01

**Authors:** Paige B. Martin, Yu Kigoshi-Tansho, Roger B. Sher, Gianina Ravenscroft, Jennifer E. Stauffer, Rajesh Kumar, Ryo Yonashiro, Tina Müller, Christopher Griffith, William Allen, Davut Pehlivan, Tamar Harel, Martin Zenker, Denise Howting, Denny Schanze, Eissa A. Faqeih, Naif A. M. Almontashiri, Reza Maroofian, Henry Houlden, Neda Mazaheri, Hamid Galehdari, Ganka Douglas, Jennifer E. Posey, Monique Ryan, James R. Lupski, Nigel G. Laing, Claudio A. P. Joazeiro, Gregory A. Cox

**Affiliations:** 1 The Jackson Laboratory, Bar Harbor, ME USA; 2 The University of Maine, Graduate School of Biomedical Science and Engineering, Orono, ME USA; 3 Center for Molecular Biology of Heidelberg University (ZMBH), DKFZ-ZMBH Alliance, Heidelberg, Germany; 4 Department of Neurobiology & Behavior, Stony Brook University, Stony Brook, NY USA; 5 Center for Nervous System Disorders, Stony Brook University, Stony Brook, NY USA; 6 Harry Perkins Institute of Medical Research, Centre for Medical Research, University of Western Australia, Nedlands, WA Australia; 7 Department of Molecular Medicine, Scripps Research, Jupiter, FL USA; 8 College of Medicine Pediatrics, University of South Florida, Tampa, FL USA; 9 Mission Fullerton Genetics Center, Mission Health, Asheville, NC USA; 10 Department of Molecular and Human Genetics, Baylor College of Medicine, Houston, TX USA; 11 Human Genome Sequencing Center, Baylor College of Medicine, Houston, TX USA; 12 Department of Genetic and Metabolic Diseases, Hadassah-Hebrew University Medical Center, Jerusalem, Israel; 13 Institute of Human Genetics, Otto-von-Guericke University Magdeburg, Magdeburg, Germany; 14 Department of Genetics, King Fahad Medical City, Riyadh, Saudi Arabia; 15 The Center for Genetics and Inherited Diseases, Taibah University, Almadinah Almunwarah, Saudi Arabia; 16 Faculty of Applied Medical Sciences, Taibah University, Almadinah Almunwarah, Saudi Arabia; 17 Neurogenetics Laboratory, UCL Queen Square Institute of Neurology, London, UK; 18 The National Hospital for Neurology and Neurosurgery, London, UK; 19 Department of Genetics, Faculty of Science, Shahid Chamran University of Ahvaz, Ahvaz, Iran; 20 GeneDx, Inc, Gaithsberg, MD USA; 21 Department of Neurology, The Royal Children’s Hospital, Melbourne, VIC Australia; 22 Murdoch Children’s Research Institute and University of Melbourne, Melbourne, VIC Australia; 23 Department of Pediatrics, Baylor College of Medicine, Houston, TX USA; 24 Texas Children’s Hospital, Houston, TX USA; 25 Department of Molecular Medicine, Scripps Research, La Jolla, CA USA

**Keywords:** Mechanisms of disease, Disease genetics, Neurodegeneration, Neuromuscular disease

Correction to: *Nature Communications* 10.1038/s41467-020-18327-6, published online 15 September 2020.

The original version of this Article contained an error in the spelling of the author Tamar Harel, which was incorrectly given as Tamar Haral. This has now been corrected in both the PDF and HTML versions of the Article.

