# Supplementary material for: NEMF mutations that impair ribosome-associated quality control are associated with neuromuscular disease
Source: Nat Commun. 2020 Sep 15;11:4625. doi: 10.1038/s41467-020-18327-6 (PMC7494853; doi:10.1038/s41467-020-18327-6)
Supplement: Supplementary file 3 — Supplementary Information [file 41467_2020_18327_MOESM3_ESM.pdf]

Supplementary Information

Martin *et al.*

NEMF mutations that impair ribosome-associated quality control are associated with neuromuscular disease

Supplementary Figure 1

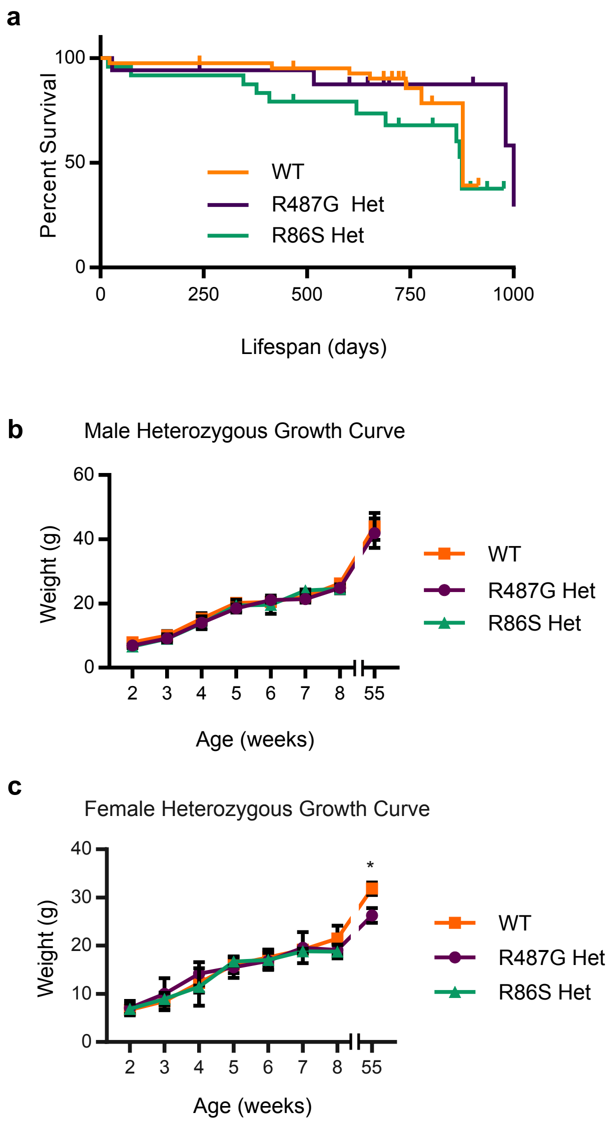

Supplementary Figure 1. Effect of heterozygous *Nemf* ENU mutations on survival and growth.

a, Kaplan-Meier survival curves of wildtype (WT) (orange line, n=42, median lifespan 877 days), R487G (purple line, n=17, 1003 days) and R86S (green line, n=24, 875 days) heterozygous mice. R86S versus WT: p=0.1415 and R487G versus WT: p=0.6389. Statistical analysis was performed by Log-rank (Mantel-Cox) test with two-tailed p value reported.

b,**c**, Body weight curves of male (b) and female (c) WT (orange square), and R487G (purple square) and R86S (green square) heterozygous mice. (n= 5,6,3 for wildtype weeks 2-4,5-7,8&55) (n=3, for 2-5 weeks, n=5 for 6 weeks, n=3 for 7 weeks and n=4 for 8 weeks R86S/+) (n=5,4,6,5 for weeks 2-3,4,5,6-55), Mean ± SD is graphed. Statistical analysis was performed by two-way ANOVA followed by S idak's multiple comparisons, non-significant at all time points except for 55-week old female R487G mice (*, p=0.0427). Source data for **b,c,** is provided in the source data file.

Supplementary Figure 2

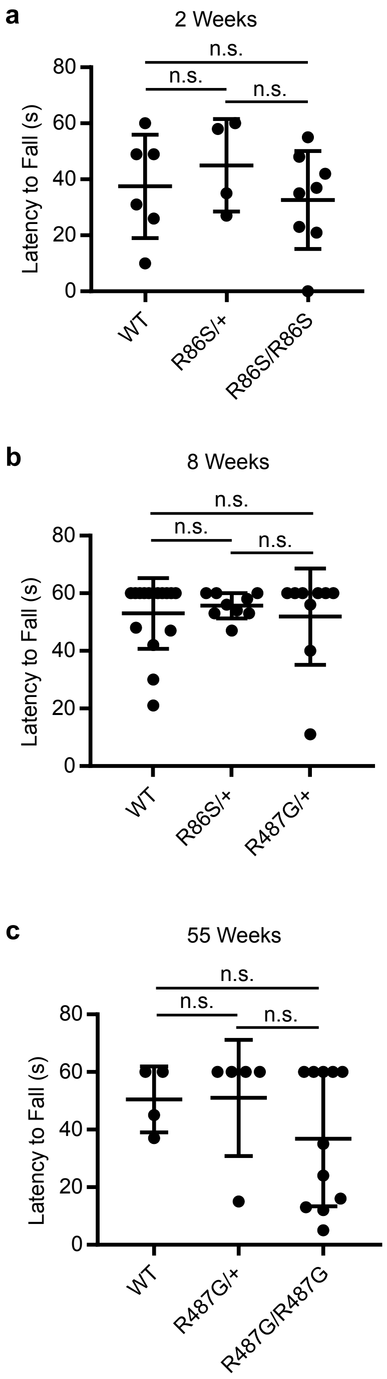

Supplementary Figure 2. Effect of homo- and heterozygous *Nemf* ENU mutations on mouse motor function.

a, Motor function of 2-week old wildtype (WT), R86S heterozygous (R86S/+) and R86S mutant (R86S/R86S) mice (n= 6, 4, and 8 respectively) measured by wire hang assay. Individual data points and mean ± SD are indicated. n.s., non-significant.

b, Wire hang duration of 8-week old WT, and R86S (R86S/+) and R487G (R487G/+) heterozygous mice (n= 16, 9, and 9 respectively). Individual data points and mean ± SD is indicated. n.s., non-significant.

c, Wire hang duration of 55-week old WT, and R487G heterozygous (R487G/+) and homozygous (R487G/R487G) mice (n= 4, 5, and 11 respectively). Individual data points and mean ± SD is indicated. n.s., non-significant. We note that the R487G homozygous mice that hang for the full 60-second duration of the experiment used only the forelimbs instead of all four limbs.

Statistical analysis was performed for all wire hang data (a-c) using a Kruskal-Wallis test followed by Dunn's multiple comparisons with adjusted p-value reported. Source data for **a,c,** is provided in the source data file.

Supplemental Figure 3

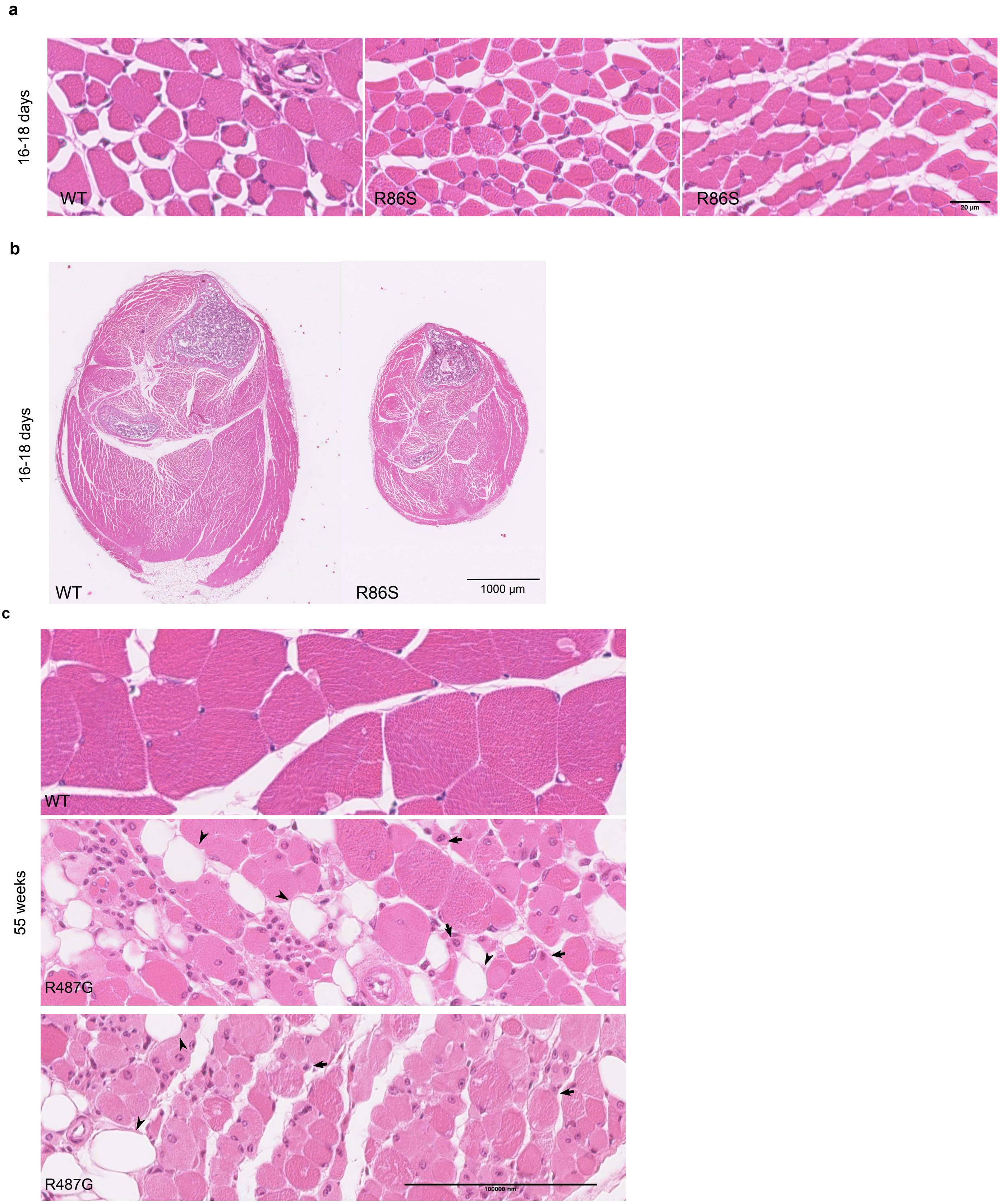

Supplementary Figure 3. Neurogenic atrophy of hindlimb muscles.

a, Histological analysis (hematoxylin-eosin staining) of medial gastrocnemius muscle (MG) in 16-18 day wildtype (WT) and two independent R86S mutants, which show smaller overall fiber size. Scale bar 20µm.

b, Cross-sections of whole lower hindlimb at 16-18days of age in shows that R86S muscles are smaller overall compared to wildtype littermate. Scale bar 1000µm.

c, Histological analysis (hematoxylin-eosin staining) of medial gastrocnemius muscle. R487G mice (two independent mice) have collagenous fibrosis (arrows) and fat depositions (arrowheads) which are not observed in WT mice. Scale bar 100 µm.

Supplementary Figure 4

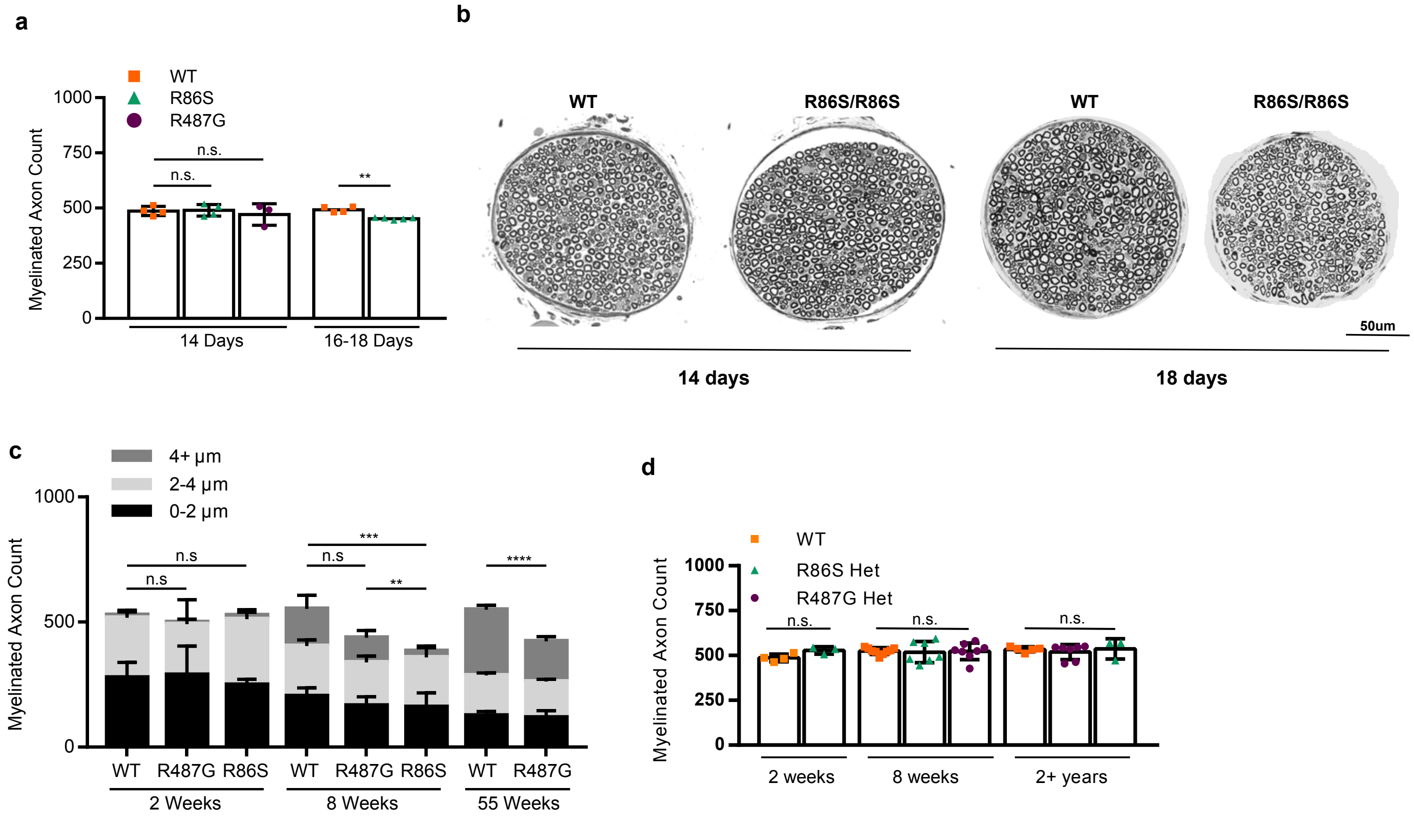

Supplementary Figure 4. *Nemf* ENU mutant mice exhibit progressive axonopathy, heterozygous do not. **a**, Myelinated axon numbers in cross-sections of femoral motor nerve branches of WT and *Nemf* ENU mice. Analysis of 2-week old wildtype (WT), R86S and R487G mice (n=4, 5 and 3 respectively), and 16-18-day old WT and R86S mice (n=4 and 5 respectively). Individual data points and mean ± SD are indicated. Statistical analysis was performed by two-way ANOVA followed by Sidak's multiple comparison, WT vs R86S (14d:p=0.9588, 16 -18d:p=0.0085), WT vs R487G (14d:p=0.8006). **b,** Representative images of WT and R86S mutants at 14 days and 18 days show a decrease in overall size of the femoral motor branch of R86S by 18 days of age and decreased myelinated axons. **c**, Numbers of small (<2 µm in diameter), medium (2-4 µm in diameter) and large (>4 µm in diameter) myelinated axons in femoral motor nerve branches. 2-week old WT, R86S and R487G homozygous mice (n=3 each), 8-week old WT, R86S and R487G homozygous mice (n=3, 4 and 5 respectively), and 55-week old WT and R487G homozygous mice (n=6 and 3 respectively) were analyzed. Mean ± SD is indicated. Statistical analysis of largest-diameter axon values was performed by one-way ANOVA followed by Tukey's multiple comparison WT vs R487G (2W: *p*>0.9999; 8W: *p*=0.1338; 55W: *p*=0.0001*);* WT vs. R86S (2W:*p*>0.9999; 8W: *p*<0.0001); R487G vs. R86S (2W: *p*>0.9999; 8W: *p*=0.0046). **d**, Myelinated axon numbers in cross sections of femoral motor nerve branches of WT and *Nemf* ENU heterozygous mice. Analysis of 2-week old WT and R86S heterozygous mice (n=4 each), 8-week old WT, R86S and R487G heterozygous mice (n=9, 7 and 8 respectively) and over 2 years of age WT, R86S and R487G heterozygous (n=5, 3 and 7 respectively). Individual data points and mean ± SD are indicated. Statistical analysis was performed by unpaired t-test (2W) or one-way ANOVA (8W. 55W), n.s., non -significant, with two-tailed p value reported. Source data for **a,c,d,** is provided in the source data file.

Supplementary Figure 5

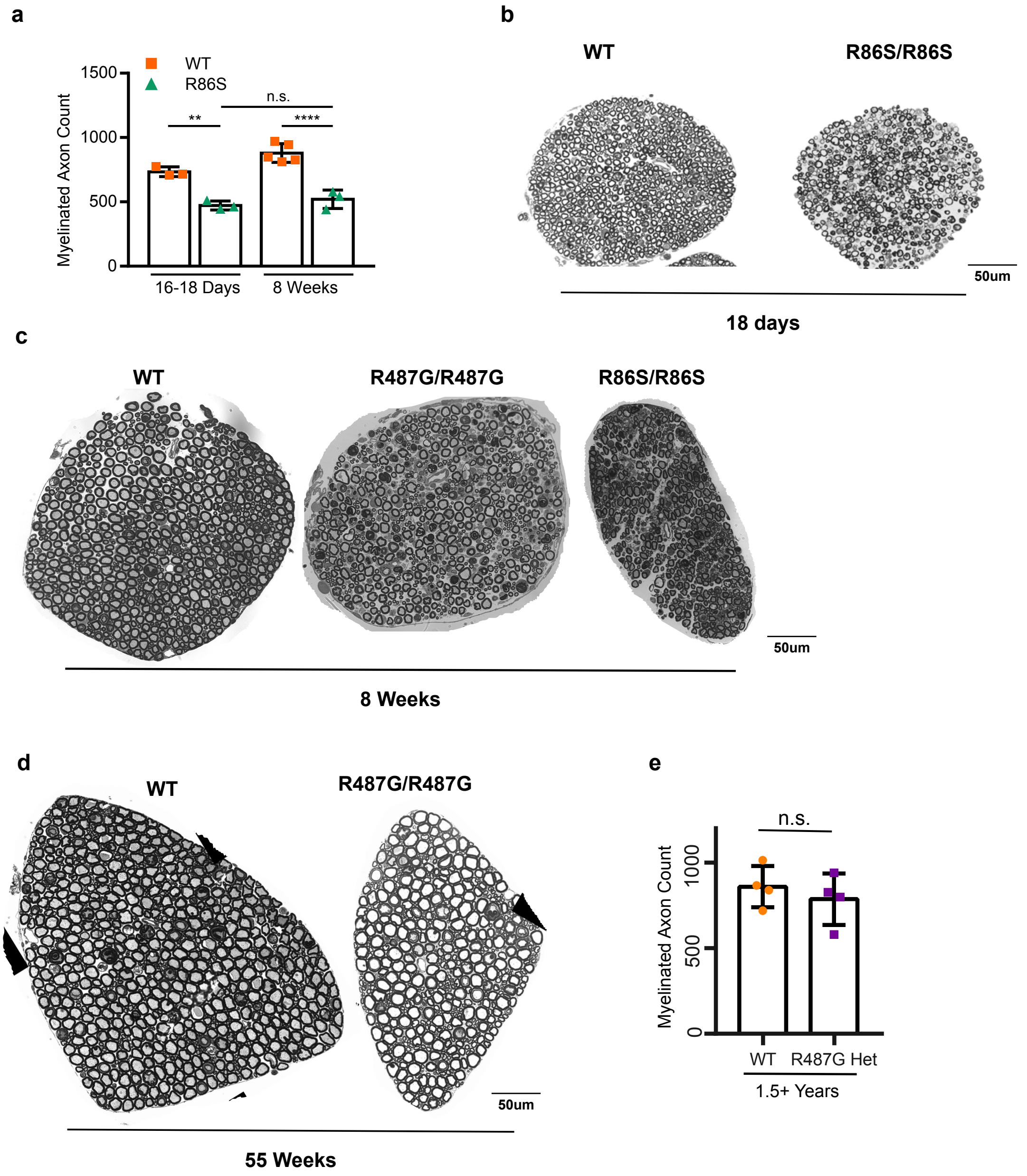

Supplementary Figure 5. *Nemf* ENU mutant mice exhibit progressive L4 ventral root degeneration, heterozygous do not.

a, Myelinated axon numbers in cross sections of L4 ventral roots WT and R86S homozygous mice. Analysis of 16-18-day old WT and R86S mice (n=3 each), and 8-week old WT and R86S mice (n=5 and 3 respectively). Individual data points and mean ± SD are indicated. Statistical analysis was performed by two-way ANOVA followed by Sidak's multiple comparison WT vs. R86S (16d: p=0.0020; 60d:p<0.0001); 16 v 60d(R86S;p=0.9230). **b,** Representative cross-sections of WT and R86S mutant L4 ventral roots at 18 days of age show a decrease in myelinated axons at 18 days of age in the mutant roots. **c,** Cross-sections of WT, R487G and R86S mutants at 8 weeks of age, show a significant reduction in size of the surviving R86S mutant's L4 roots compare to WT or R487G at the same timepoint. **d,** Cross-sections of L4 ventral roots of WT and R487G mutants at 55 weeks of age indicate a reduction in size and number of myelinated axons remaining in the R487G mutants. **e**, Analysis of WT (n=4) and R487G heterozygous (n=4) L4 ventral root myelinated axon counts indicates that even at 1.5 year of age or older these mice do not display a degeneration in myelinated axons. Individual data points and mean ± SD are indicated. Statistical analysis was performed by an unpaired t-test with two-tailed p=0.4799, n.s., non-significant. Source data for **a,e,** is provided in the source data file.

Supplementary Figure 6

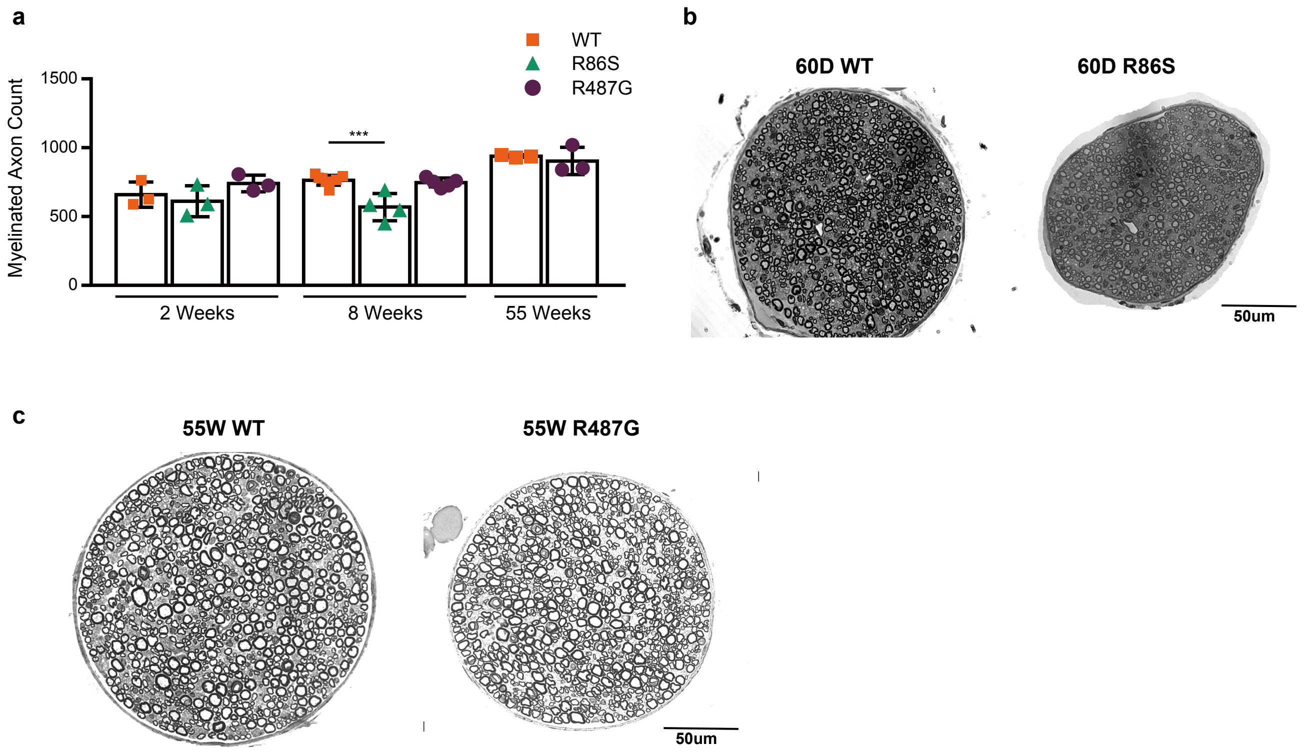

Supplementary Figure 6. *Nemf* R86S mutant mice exhibit sensory axonopathy, R487G does not.

a, Myelinated axon numbers in cross sections of femoral sensory nerve branches of WT and *Nemf* ENU homozygous mice. Analysis of 2-week old WT, R86S and R487G mice (n=3 each), 8-week old WT, R86S and R487G mice (n=9, 4 and 5 respectively), and 55-week old WT and R487G mice (n=3 each). Individual data points and mean ± SD are indicated. Statistical analysis was performed by two-way ANOVA followed by Sidak's multiple comparison, WT vs R86S 8W (p=0.0007).

b, Cross-section of the sensory branch of the femoral nerve at 8 Weeks of age shows a significant reduction in overall size and myelinated axons in the R86S mutant sensory branch.

c, Cross-sections of the sensory branch in the 55 weeks of age wildtype and R487G mutants shows no significant difference in size or myelinated axons present. Source data for **a** is provided in the source data file.

Supplementary Figure 7

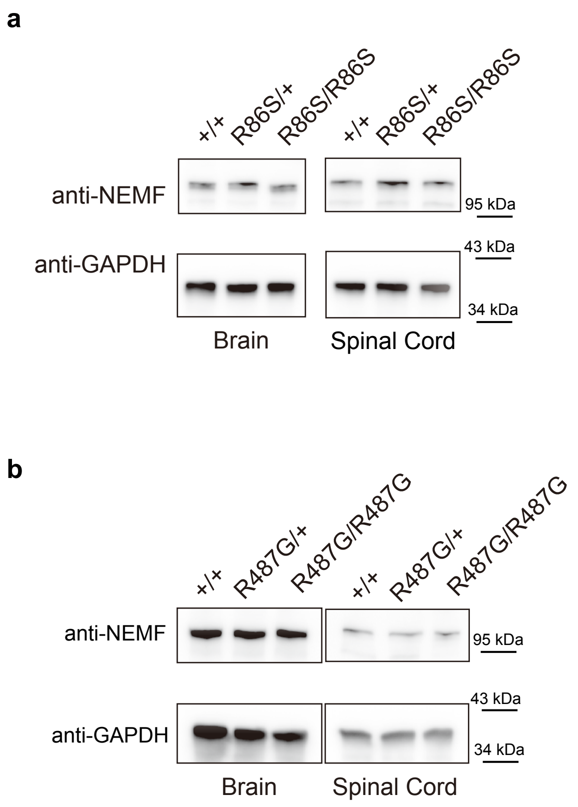

Supplementary Figure 7. NEMF protein levels are not affected in *Nemf* ENU mutant mice.
a, Western blot analyses of brain and spinal cord lysates from 45-day old wildtype, +/R86S heterozygous, and R86S homozygous mice, immunoblotted with anti-NEMF antibody (and with anti-GAPDH antibody as loading control).
b, Western blot analyses of brain and spinal cord lysates from 65-day old wildtype, +/R487G heterozygous and R487G homozygous mice, immunoblotted with anti-NEMF antibody (and with anti-GAPDH antibody as loading control). Source data is provided in the source data file. Associated uncropped blots (Supplementary Fig. 11).

Supplementary 8

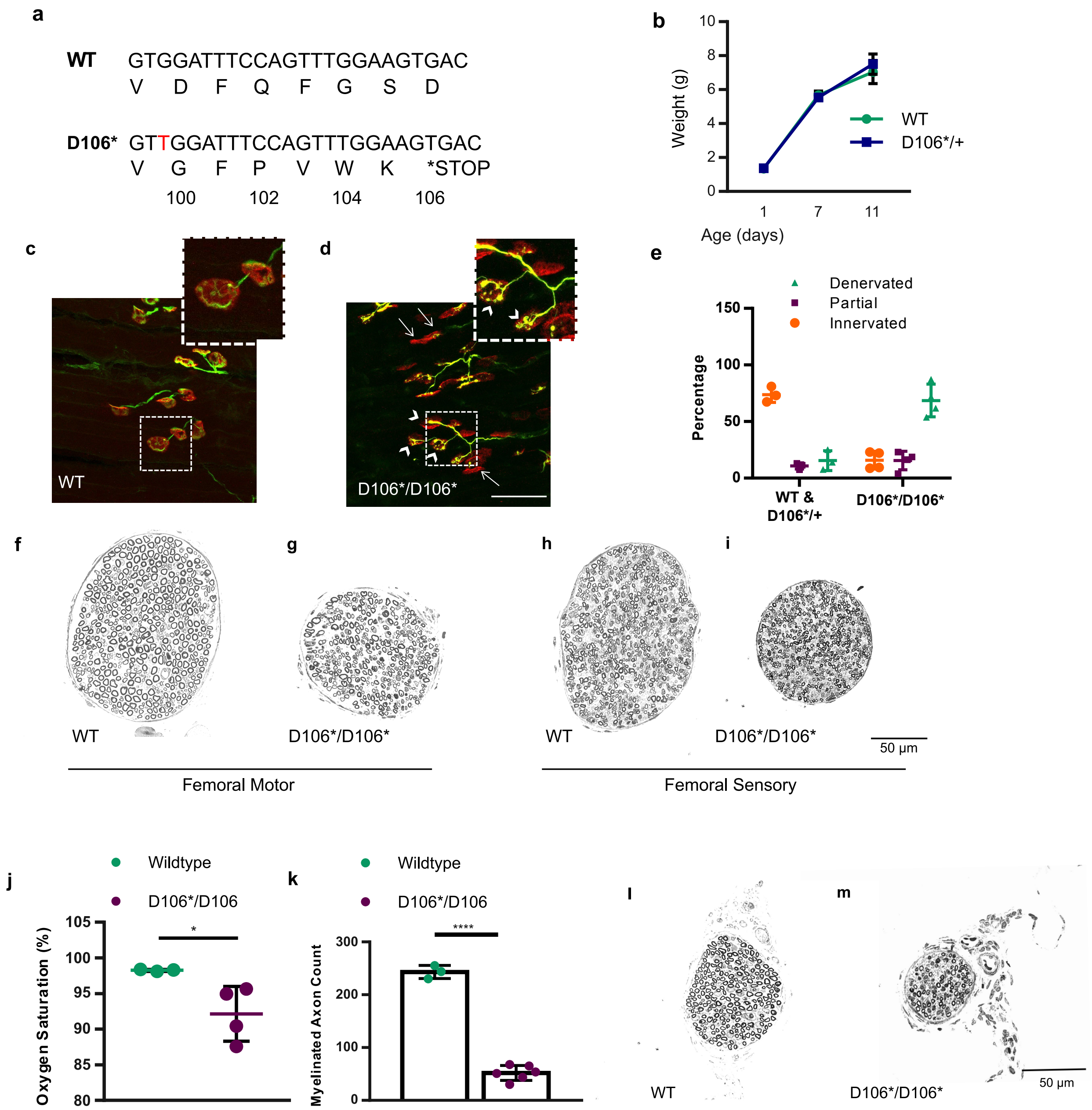

Supplementary Figure 8. *Nemf* D106* mice display perinatal neurodegeneration.
a, Schematic diagram of the CRISPR-*Cas9*-induced mutation in the *Nemf* D106* strain (caused by a T insertion at nucleotide position 295 in the open reading frame, indicated by a red T in the D106* sequence). Single nucleotide insertion causes a frameshift leading to premature termination at Asp106. Amino acid positions are indicated. **b**, Body weights of *Nemf* D106* heterozygous (blue squares, n=9, 7 and 3 on days 1, 7 and 11 respectively) and WT mice (green circles, n=8, 5 and 5 on days 1,7 and 11 respectively) . Mean ± SD is indicated. Statistical differences were analyzed by two-way ANOVA, and were non-significant at all time points. **c-d**, Neuromuscular junctions (NMJs) of medial gastrocnemius muscle from 10-day old WT and *Nemf* D106* homozygous mice. Tissues were stained for pre-synaptic vesicles (anti-SV2, green), neurofilaments (anti-neurofilament 2H3, green) and acetylcholine receptor (α-bungarotoxin, red). *Nemf* D106* homozygous mice have partially occupied (arrowhead) and unoccupied (arrow) junctions that are not observed in WT mice. Scale bar 130μm. Inset scale bar 65 μm. **e**, NMJ occupancy. Quantification of fully innervated, partially innervated or denervated junctions based on the pre- and post-synaptic staining overlap. Mean ± SD is indicated. Statistical analysis was performed by two-way ANOVA followed by Sidak's multiple comparison Unaffected vs. Hom (denervated:$p<0.0001$;partial: $p=0.8784$; innervated: $p<0.0001$). **f-g**, Cross-sections of femoral motor branches of 10-day old wildtype and *Nemf* D106* homozygous mice. Scale bar, 50 μM. **h-i,** Cross-sections of femoral sensory nerve branches of 10-day old wildtype and *Nemf* D106* homozygous mice. Scale bar, 50 μM. **j,** PulseOx analysis of D106*/D106* mutants (92 ± 4, n=4, purple circles) shows a significantly lower oxygen saturation (%) than wildtype littermates (98 ± 0.1, n=3, green circles). Individual data points and mean ± SD are indicated. Statistical analysis was performed by upaired t-test, with two-tailed $p=0.0428$). **k,** Wildtype (green circles) littermates have significantly more myelinated axons in the phrenic nerve (243 ± 13, n=3) compared to the NEMF-null mutant (purple circles) phrenic nerve (52 ± 14, n=6, p<0.0001). Individual data points and mean ± SD are indicated. Statistical analysis was performed by unpaired t-test, with a two-tailed $p<0.0001$. **l-m,** Phrenic nerve cross section showing myelinated axons and overall nerve size in wildtype (l) and NEMF-null mice (m). Scale bar, 50μm. Source data for **b,e,j,k** is provided in the source data file

Supplementary Figure 9

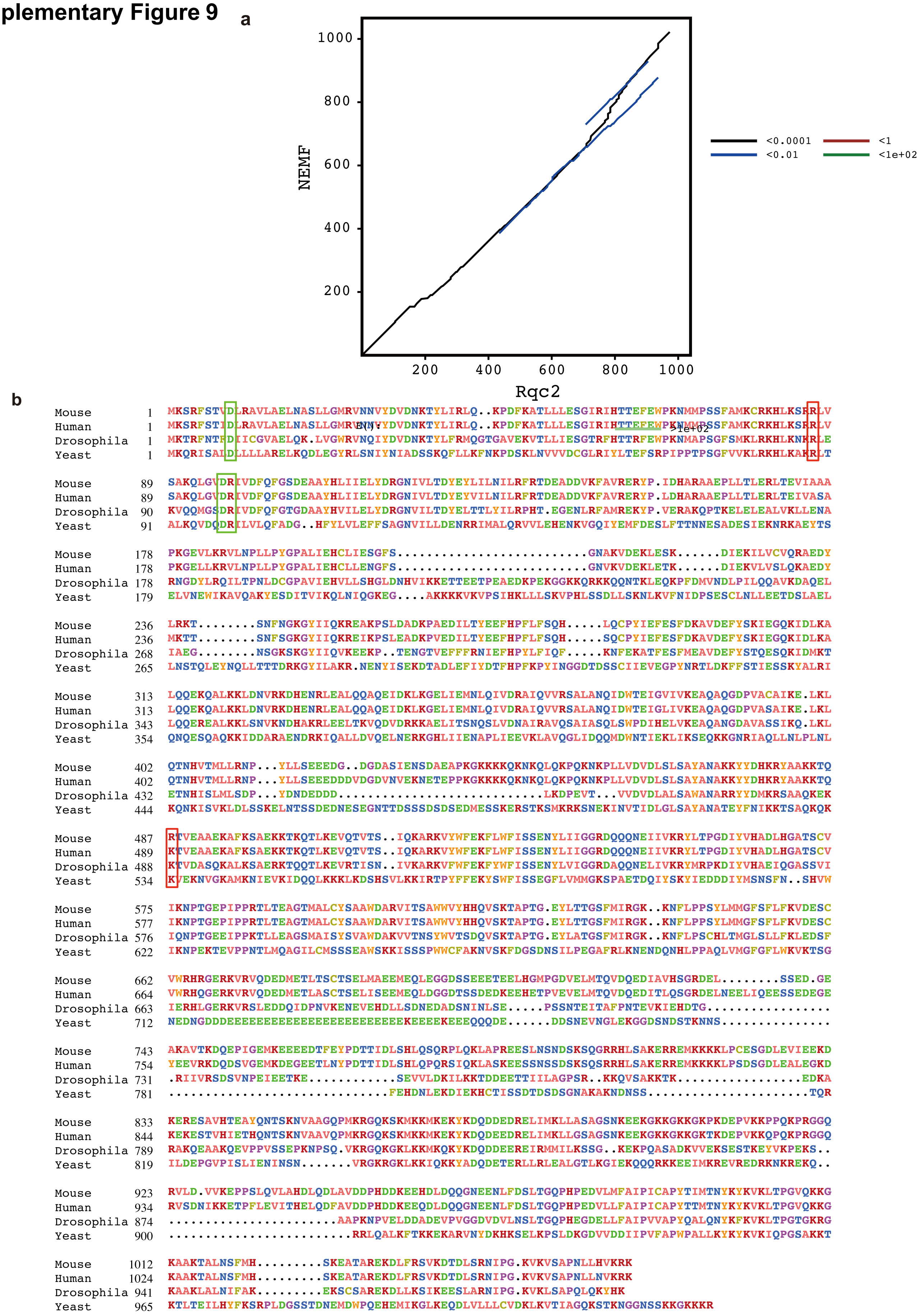

Supplementary Figure 9. Residues affected by the ENU mutations are conserved between mouse NEMF and its yeast Rqc2 homolog.

a, Multiple sequence alignment of mouse NEMF and yeast Rqc2 was generated, followed by sequence similarity plotting using PLALIGN software.

b, Multiple sequence alignment of NEMF homologues in mouse, human, fly (Caliban) and yeast (Rqc2). Mouse NEMF Arg86 and Arg487 affected by the ENU-induced mutations (red rectangles), and yeast Rqc2 Asp9, Asp98 and Arg99 (green rectangles) implicated in CAT tail synthesis are highly conserved across organisms. Asp98 is mutated to Tyr in the CATylation -deficient Rqc2 D98Y mutant construct, and Asp9, Aps98 and Arg99 are mutated to Ala in the Rqc2 aaa mutant construct.

Supplementary Figure 10

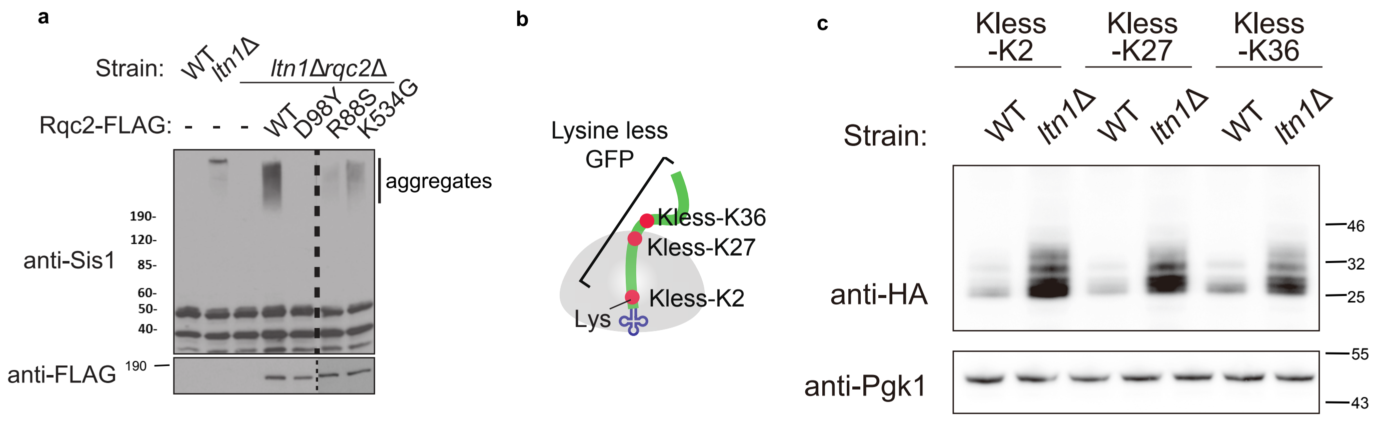

Supplementary Figure 10. ***Nemf*** **ENU mutations selectively affect the CATylation function of Rqc2.**

a, Strains were transformed with Rqc2-FLAG constructs, as indicated. Anti-Sis1 blot monitored endogenous aggregates.

b, Diagram of the CAT tail-dependent degradation reporter (HA-tagged Kless-K2). This reporter completely lacks Lys residues, except at two positions buried well into the ribosomal exit tunnel, 2 residues N-terminal to the stalling sequence and ~35 residues away from the tunnel opening. Thus, CAT tail synthesis is required for those Lys residues to become exposed to Ltn1 for ubiquitination. The Lys residues in the Kless-K27 reporter are positioned closer to the opening of the exit tunnel, whereas the Lys residues in the Kless-K36 are predicted to be already exposed on the outside.

c, Kless-K2, -K27 and -K36 reporter expression in wildtype (WT) or Ltn1-deficient (*ltn1Δ*) cells. Cell lysates were immunoblotted with anti-HA antibody to monitor reporter expression, and anti-Pgk1 as loading control.

Supplementary Figure 11 Uncropped Original Scans

Figure 3a

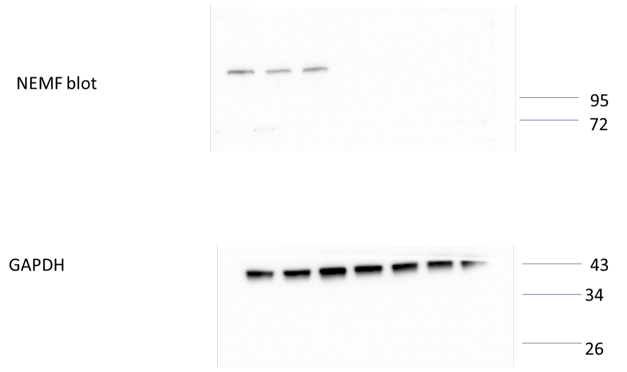

Figure 4b

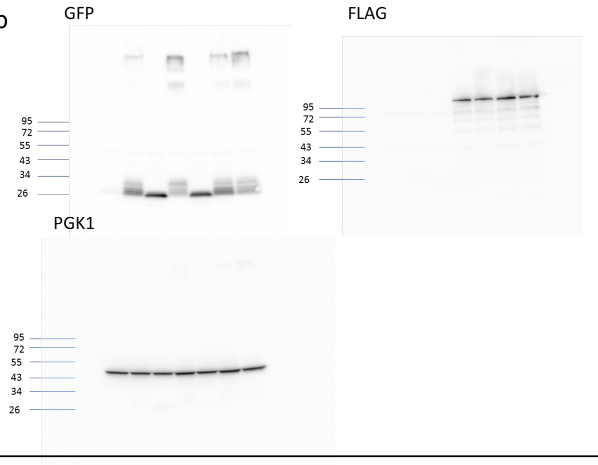

Figure 4c

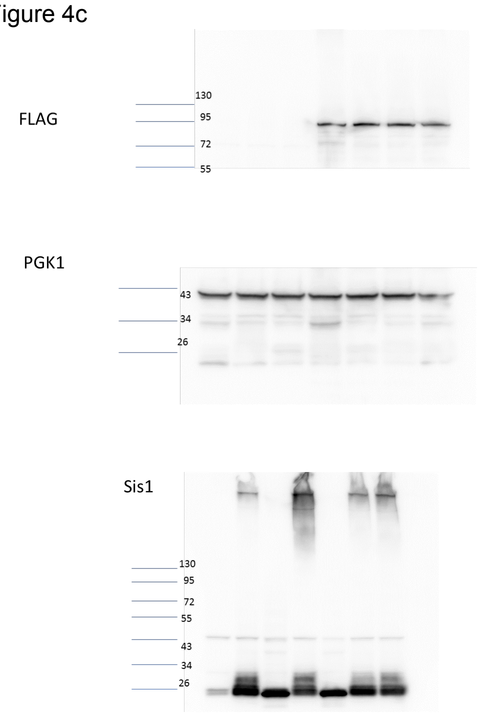

Figure 4d

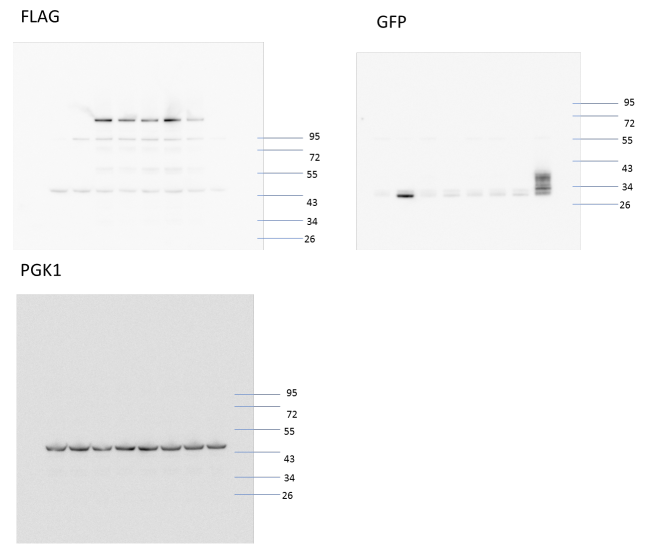

Figure 4e

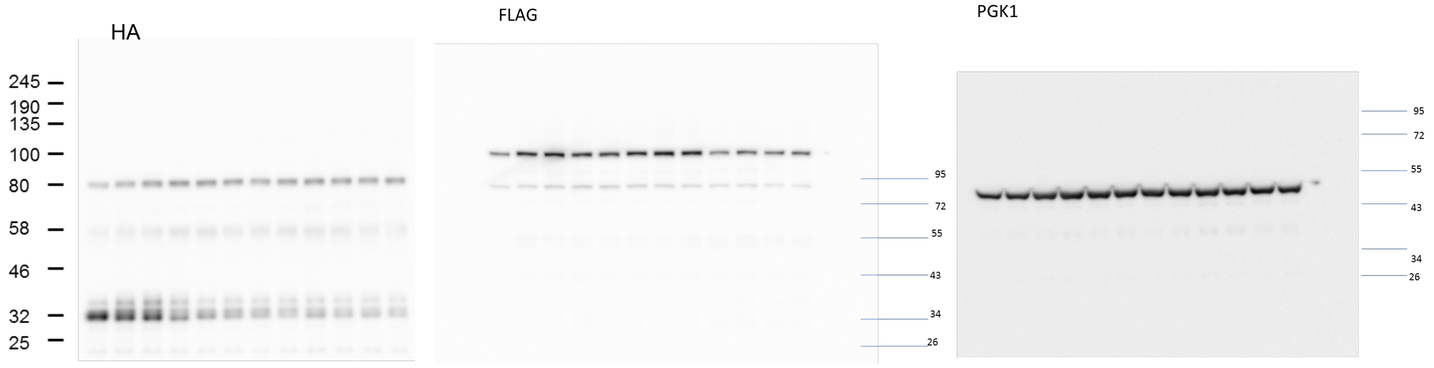

Supplementary Figure 12. uncropped blots and molecular weights

Supp. 7a uncropped blots

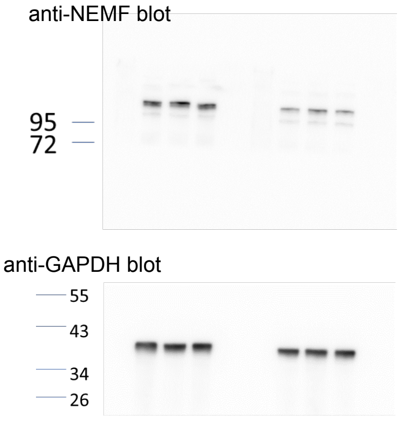

Supp. 7b uncropped blots

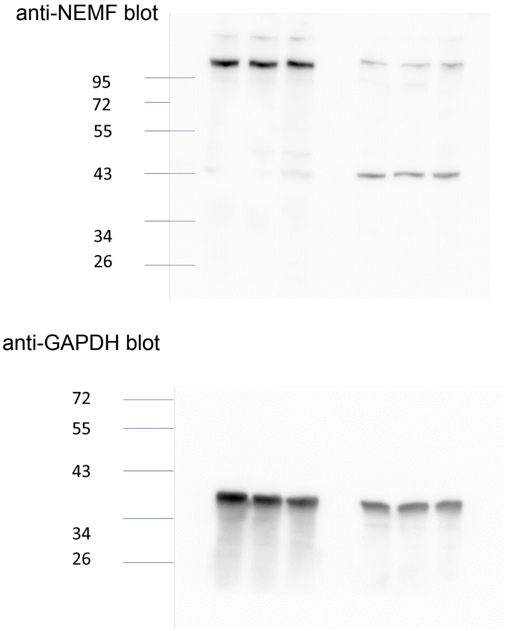

Supp. 10a uncropped blots

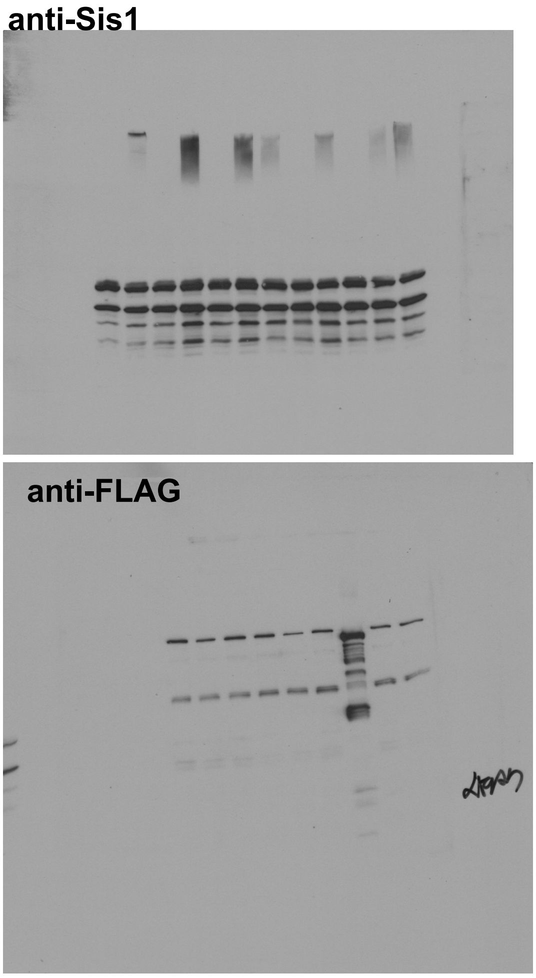

Supp. 10c uncropped blots

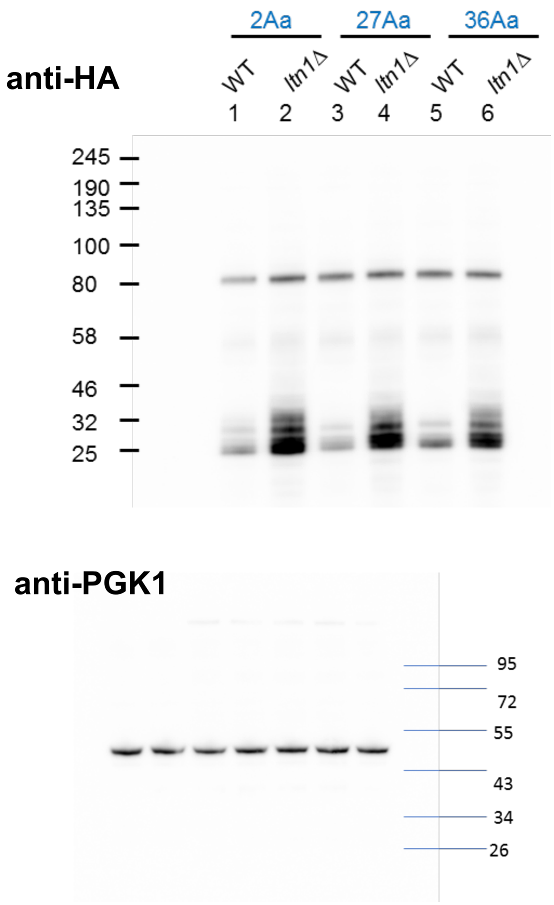

Supplementary Note 1: clinical summaries

For all patients informed consent was obtained to be included in the study and have clinical information published.

AUS1

This patient is the third born child (AUS1-II:3) to a consanguineous Syrian couple. At 18 months of age it was noted that he had delayed motor and language development, with increased tone and clonus in the lower extremities, but with decreased reflexes which suggested a concomitant neuropathy. He had some dysmorphism, including bilateral thumb adduction abnormalities and pectus carinatum. He stood independently at age 3, at which time marked equinovarus posturing of the distal lower extremities was apparent. Nerve conduction studies (NCS) at age 4 years showed low amplitude tibial and absent peroneal responses bilaterally, with normal sural sensory studies. Electromyography (EMG) of the tibialis anterior (TA) and medial gastrocnemius (MG) showed mild chronic neurogenic changes, while EMG of the first dorsal interosseous was normal. Cardiac and metabolic screening examinations were normal. Due to the consanguineous nature of the patient, inherited disease was suspected. Screening for a molecular genetic cause indicated that the patient was normal for a SNP microarray testing panel, did not have Fragile X syndrome, and did not identify any likely pathogenic variants on a neurogenetic disease genetic panel (including 376 genes associated with nerve disorders). Whole genome sequencing (WGS) was performed through the Australian Genomic Health Alliance (AGHA) neuromuscular flagship and the data analyzed in seave (KCCG, Garvan Institute). WGS indicated a homozygous nonsense variant (c.2608C>T; p.Arg870*) in *NEMF*.

DEU1

The patients were the third and sixth born children of a healthy Turkish couple. Parents denied consanguinity. None of the four unaffected children had any developmental or neurological problems. The older affected male sibling (DEU1-II:3) was diagnosed with an atrial septal defect at birth (spontaneous closure by the age of 2) and had a rudimentary extra digit attached to the base of the right thumb (preaxial polydactyly). Global developmental delay was noted at the age of 2 years when he had just started walking and was able to speak less than 5 words. He received speech and occupational therapy and made continuous developmental progress. Repeated assessment of his intellectual capacities showed global impairments in the range of mild intellectual disability. Neurologic evaluation at the age of 7 documented poor coordination but no focal neurologic deficits.

Parents reported slowly progressive gait abnormalities with onset at about 10 to 12 years old. At age 14 he had significant weakness of dorsiflexion (foot drop) of both feet with mild paresis of plantarflexion and muscular atrophy of lower legs. Sensory functions were not obviously impaired. His gait was mildly ataxic. Tendon reflexes of the lower extremities were brisk. Mild articulation difficulties were noted. Nerve conduction studies at age 14 years showed absent responses for N. peroneus and N. suralis and low amplitude for the N. tibialis bilaterally, with a tibial nerve conduction velocity (NCV) of 29 m/s. Electromyography (EMG) of the tibialis anterior muscle showed chronic neurogenic changes. Together, electrophysiology findings were suggestive of a mainly axonal neuropathy. He developed progressive thoracal kyphosis with mild scoliosis and lumbar hyperlordosis. The knees were in moderate valgus position. Cranial MRI at the age of 17 showed mildly enlarged internal and external fluid spaces (suggesting mild cerebral atrophy), a few small and non-specific white matter lesions and a small left frontal arachnoid cyst. His body measurements at the age of 17 were within the normal ranges: weight 82 kg (+1.2 SD), height 172 cm (-0.4 SD, head circumference 57.7 cm (+0.7 SD).

The younger affected female sibling (DEU1-II:6) had a very similar phenotype but apparently more severe neurological impairment. Muscular hypotonia and feeding difficulties were noted in infancy. Developmental progress was slow. Abnormal foot posture with a bilateral non-fixed clubfoot was first noted at the age of 1, and muscular atrophy of lower legs was documented during follow-up. Despite the progressive atrophy the tendon reflexes were rather brisk. A systolic murmur led to the diagnosis of a small atrial septal defect that was closed at a control echocardiography at the age of 3. A cranial MRI at the age of 1.5 years was normal. She learned to walk without support about the age of 3.5 years. Speech development was also significantly delayed. Electrophysiology at ages 2.5 and 5 years documented a progressive mainly axonal neuropathy with a decrease loss of peroneal responses bilaterally. Together, clinical and neurophysiological findings were considered suggestive of not just peripheral neuropathy but also some central motor involvement. She was diagnosed with mild to moderate intellectual disability without obvious regression of cognitive functions. At last examination at the age of 11.2 years her body measurements were: weight 27 kg (-2.7 SD), height 128 cm (-2.2 SD), head circumference 49.1 cm (-3.5 SD; apparent decline of head growth from -2.0 SD at age 1.5 years). She had a slim built, severe muscle atrophy of lower legs and truncal hypotonia. Despite significant muscular atrophy of lower extremities, Achilles tendon reflexes were responsive and patellar tendon reflexes were brisk. Her gait was uncoordinated and ataxic with prominent foot extensor paresis. There was mild weakness of upper extremities, no tremor or dysmetria. She also had severe kyphoscoliosis, scapulae allatae and genua valga.

Diagnostic whole exome sequencing was performed on leukocyte DNA from both affected siblings. In both a homozygous nonsense variant (c.2014A>T; p.(Lys672*)) in *NEMF* was identified. Both siblings shared an approximately 9 Mb interval of homozygosity at the *NEMF* locus, thus indicating inheritance from a common ancestor. The variant is classified as likely pathogenic according to ACMG criteria [1].

IRN1

This patient is a 28-years-old boy (IRN1-II:1) with three healthy sisters born to unaffected first-cousin Persian parents. After 36 weeks of pregnancy, he was born via prolonged and severe vaginal delivery and presented with hypotonia at birth. He had delayed neck control and could not sit unsupported until nine months of age. He started walking with help at the age of 18 months and displayed progressive abnormal gait with poor coordination, which at the age of 8 years was noticed. He had speech delay, failing to ever to develop language and presently presents with limited expressive language. His cognition is below normal, even though the IQ level has not been measured officially. Although he can do some of day-to-day life activities, he need support with others such as taking bath and he went to school with special education for only 6 years. He also gradually developed distal muscle atrophy and claw hands and as a result of this he cannot use pen and write. Additional clinical feature is strabismus. His growth is within normal range, and he doesn't have any behavioral abnormalities. No clinical investigation such as EMG, brain MRI, metabolic assay was performed. Whole genome sequencing (WGS) was performed on DNA from the proband and data analysis indicated a novel homozygous frameshift variant (c.2451delA; p.(Ser817*fs**7)) in NEMF residing within a ~7 Mb region of homozygosity. The variant was not identified in the in-house database consisting of 14000 exomes.

SAU1

These patients are two brothers both born preterm to a healthy 1st cousin parent. The index (SAU1:II-1) is a 10.5 year-old male, born preterm at 34 weeks NSVD with history of NICU admission for prematurity and transient respiratory disease that needed no ventilatory support. He has motor and cognitive delay, including all milestones noted within the 1st year of life. He sat at 2 year, stood at 2.5 year and walked at 3.5 years. At 4 years of age, he was noted to have special attention to moving subjects, more hyperactive with very retarded speech and cognitive functions and unable to control sphincters. Upon neurological assessment, he was found to have attention deficit hyperactivity disorders (ADHD) with subclinical seizures (abnormal EEG) that needed antiepileptic medication (Depakane). Neuro-behavioral assessment at 8 years showed that he can complete sentences comprised of 3 words only and is only able to understand simple commands. Speech assessment

showed moderate receptive language delay and severe expressive delay with phonological errors. The overall IQ score was 81. He had normal vision and hearing. He was referred to endocrine specialty because of distinctive signs of precocious puberty in form of increased facial hair and relatively large phallus. However, hormonal assay for thyroid, growth hormone, LH/FSH and testosterone analyses were unremarkable. Assessment at 10.5 years of age, revealed weight of 34.75 kg (50th), height 135 cm (25th) and OFC 52.2 cm (50th). He has experienced no seizures, but remains hyperactive with overt social interaction, very talkative behavior and, now, maintained good sphincter control. He is unable to run and standing from sitting position. He has difficulities in holding pen and drawing circles and straight lines. He has increased facial hair with no facial dysmorphia. Neurological assessment and systemic examination are unremarkable. Genital examination showed normal-sized phallus with normal testes. The brain CT and MRI done at 4 years and both reported as normal. EEG showed generalized nonspecific epileptic waves with left frontal discharges. He is currently in a modified learning environment with marked learning delay, especially in mathematics and simple science. However, he showed very dramatic improvement in simple memory and writing skills.

The other younger affected sib (SAU1:II-2) is 7-year-old born preterm NSVD. The birth parameters were not recalled. However, there were no maternal risk factors. The parent noted delayed motor milestone from the second year of life. But he gained motor skills at 5 years. Now, he has difficulty in running. At 4 years, he was diagnosed with ADHD with speech dysfunction. Neurodevelopmental assessment using Vineland adaptive behavior scales showed communication score of 54; moderate deficit, socialization 69; mild deficit, daily living skills 41; moderate deficit and composite score of 50, indicating moderate deficit. Brain MRI done at 3.5 years, showed normal myelination and cerebral and cerebellar structures. Clinical examination at 6.5 year showed normal weight (18.25 kg), height (110 cm) and head circumference (48 cm). There were no facial dysmorphia and other systemic examination was unremarkable, apart from hexadactyly of the right hand.

Tandem MS and urine organic acid screening were unremarkable. Array CGH didn't show any gain or loss. The Whole Exome Sequencing in the affected siblings showed a homozygous frameshift variant in *NEMF* (c.2871_2875dupTGTAG; p.(Asp959fs*2)) . Parents were heterozygous for the same mutation. The healthy sibling was heterozygous for this variant.

USA1

Patient USA1-II:1 is the first-born to healthy, non- consanguineous parents. She had normal early childhood development, attaining age-appropriate motor and intellectual developmental milestones: rolling by 1-2 months of age, sitting by 5 months, and first words by 1 year of age. The patient first presented with Chiari malformation which was surgically corrected at one and a half years of age. Post-operatively the patient required full-time ventilation due to respiratory failure. The patient is currently wheelchair-bound and requires 24/7 respiratory ventilation and G-tube dependence. The patient has received speech and occupational therapy. Whole exome sequencing revealed multiple mutations in *NEMF*, a *de novo* variant of the start codon (c.1A>T; p.(Met1Leu)), and a maternally inherited nonsense variant (c.2011C>T; p.(Arg671*)). It cannot be ruled out that the *de novo* and maternally inherited variants are on the same allele, as phase has not been determined.

USA2

Patient USA2-II:1 is a first-born female to non-consanguineous parents. She was reported to have experienced gastrointestinal illness-related non-febrile seizures at three and four years of age, resulting in the request for a brain MRI at four years of age, which indicated delayed myelination. Additionally, she presents/ed with mild hypotonia and significant bouts of clumsiness, as well as difficulty with both gross and fine motor coordination leading to falls which have caused severe lacerations. In addition to motor deficits, the patient has been diagnosed with Autism Spectrum Disorder and displays mild intellectual disability with some speech articulation difficulties. Behavioral changes around 2 years of age include OCD like-behaviors and sensitivities to loud noises. Neurologic evaluation at the age of 5 years and 8 months documented poor coordination but no focal neurologic deficits. Genetic testing was requested in order to determine if these findings were a metabolic disorder. Metabolic screening determined no variations, however whole exome sequencing indicated two maternally-inherited missense mutations in *NEMF* (c.980 G>A; p.Arg327Gln and c.2777C>T; p.(Pro926Leu)) and a paternally-inherited frameshift variant (c.2768delA; p.(Lys923Arg*fs**27)). The c.980 G>A, c.2777C>T and c.2768delA variants are present on five, one and two alleles, in gnomAD, respectively.

USA3

Patient USA3-II:1 initially presented at the age of 17 due to new onset tremor for about 10 months and unable to walk on his heels. On further questioning, he was found to be clumsy throughout his life, a bit slower compared to his peers and has had several unexplained falls. However, he was involved in contact sports including rugby, soccer and basketball. He also received speech therapy from 3 to 5 years but always making good school grades. Physical

examination at the age of 19 was remarkable for bilateral distal muscle atrophy in lower limbs, pes cavus, decreased muscle strength (4/5) in dorsiflexion of feet. Reflexes were diffusely diminished (1+) in upper and ankle reflexes were not obtained. He had a steppage gait and unable to perform heel walking. Bilateral fine tremor in hands was noticeable without any additional cerebellar findings. CK was mildly elevated 379 U/L (N: 55-370) and EMG/NCS showed axonal motor and sensory neuropathy. Clinical exome sequencing on the index was unremarkable. Expansion to trio exome (index-mother-father) as part of Baylor-Hopkins Center for Mendelian Genomics research initiative revealed a *de novo* missense variant in *NEMF* (c.1658T>C, p.(Ile553Thr)). This variant was absent from gnomAD and affects an amino acid that is conserved to yeast (Supp. Fig 8b). Based on the ACMG guidelines for variant interpretation, a *de novo* change in a gene compatible with the observed phenotype, is classified as Class 4 and likely pathogenic [1].

Supplementary References

1. Richards, S. *et al.* Standards and guidelines for the interpretation of sequence variants: A joint consensus recommendation of the American College of Medical Genetics and Genomics and the Association for Molecular Pathology. *Genet. Med.* **17**, 405–424 (2015).
