# Supplementary material for: NEMF mutations that impair ribosome-associated quality control are associated with neuromuscular disease
Source: Nat Commun. 2020 Sep 15;11:4625. doi: 10.1038/s41467-020-18327-6 (PMC7494853; doi:10.1038/s41467-020-18327-6)
Supplement: Supplementary file 5 — Description of Additional Supplementary Files [file 41467_2020_18327_MOESM5_ESM.pdf]

<u>Description of Additional Supplementary Files</u>

Title: Supplementary Movie 1.

Description: R86S mice display paddle-like gait at 60 days of age. R86S male mouse with wildtype sibling at 60 data of age displays hindlimb wasting and abnormal gait.

Title: Supplementary Movie 2.

Description: Aged R487G mice display abnormal gait. R487G male mouse at ~260 days of age displays hindlimb wasting and waddling gait, compared to unaffected wildtype littermate.
