# Supplementary material for: NEMF mutations that impair ribosome-associated quality control are associated with neuromuscular disease
Source: Nat Commun. 2020 Sep 15;11:4625. doi: 10.1038/s41467-020-18327-6 (PMC7494853; doi:10.1038/s41467-020-18327-6)
Supplement: Supplementary file 6 — Reporting Summary [file 41467_2020_18327_MOESM6_ESM.pdf]

nature research

Corresponding author(s): Gregory A. Cox

Last updated by author(s): 06-24-2020

Reporting Summary

Nature Research wishes to improve the reproducibility of the work that we publish. This form provides structure for consistency and transparency in reporting. For further information on Nature Research policies, see Authors & Referees and the Editorial Policy Checklist.

Statistics

For all statistical analyses, confirm that the following items are present in the figure legend, table legend, main text, or Methods section.

n/a	Confirmed	
☐	☒	The exact sample size (*n*) for each experimental group/condition, given as a discrete number and unit of measurement
☐	☒	A statement on whether measurements were taken from distinct samples or whether the same sample was measured repeatedly
☐	☒	The statistical test(s) used AND whether they are one- or two-sided<br>*Only common tests should be described solely by name; describe more complex techniques in the Methods section.*
☒	☐	A description of all covariates tested
☒	☐	A description of any assumptions or corrections, such as tests of normality and adjustment for multiple comparisons
☐	☒	A full description of the statistical parameters including central tendency (e.g. means) or other basic estimates (e.g. regression coefficient) AND variation (e.g. standard deviation) or associated estimates of uncertainty (e.g. confidence intervals)
☐	☒	For null hypothesis testing, the test statistic (e.g. *F*, *t*, *r*) with confidence intervals, effect sizes, degrees of freedom and *P* value noted<br>*Give P values as exact values whenever suitable.*
☒	☐	For Bayesian analysis, information on the choice of priors and Markov chain Monte Carlo settings
☒	☐	For hierarchical and complex designs, identification of the appropriate level for tests and full reporting of outcomes
☒	☐	Estimates of effect sizes (e.g. Cohen's *d*, Pearson's *r*), indicating how they were calculated

Our web collection on statistics for biologists contains articles on many of the points above.

Software and code

Policy information about availability of computer code

Data collection	For data collection the softwares: FIJI (ImageJ1.52p), NDP.View2((U12388-21, Hamamatsu), STRAP web-based software(http://www.bioinformatics.org/strap/aa/), ClustalW (version 1.2.2.).
Data analysis	For data analysis GraphPad Prism version 7 for windows was used.

For manuscripts utilizing custom algorithms or software that are central to the research but not yet described in published literature, software must be made available to editors/reviewers. We strongly encourage code deposition in a community repository (e.g. GitHub). See the Nature Research guidelines for submitting code & software for further information.

Data

Policy information about availability of data

All manuscripts must include a data availability statement. This statement should provide the following information, where applicable:

- Accession codes, unique identifiers, or web links for publicly available datasets
- A list of figures that have associated raw data
- A description of any restrictions on data availability

Data associated with are available from the respective websites: GeneDx ClinVar submission page (http://www.ncbi.nlm.nih.gov/clinvar/submitters/26957/), Gnomad database (https://gnomad.broadinstitute.org/), GME Variome (http://igm.ucsd.edu/gme/), Iranome (http://www.iranome.ir/) and 1000 Genomes Project (https://www.internationalgenome.org/).

Field-specific reporting

Please select the one below that is the best fit for your research. If you are not sure, read the appropriate sections before making your selection.

☒ Life sciences ☐ Behavioural & social sciences ☐ Ecological, evolutionary & environmental sciences

For a reference copy of the document with all sections, see nature.com/documents/nr-reporting-summary-flat.pdf

Life sciences study design

All studies must disclose on these points even when the disclosure is negative.

Sample size	Sample sizes of mice were chosen by comparison to previously published neuromuscular disease models in the lab (SMARD1 model: Cox et al. 1998;Grohmann et al, 2004), Listerin:Chu et al 2009) to achieve statistical significance for body weight, wire hang and histological phenotypes. Some later time-points for severely affected mice (8 weeks R86S mutants) had only 3 biological replicates due to early disease-related lethality.
Data exclusions	no data was excluded.
Replication	Independent cohorts of wild-type and mutant mice were used to replicate the onset and progression of symptoms used for analyses. A minimum of 3 litters were observed per time-point for each measure reported (axon analysis, weights, lifespan, oxygen saturation, nmjs, H & E). NMJ staining was replicated with 3 different cohorts, each with Wildtype, Heterozygous and Mutant littermates to control for antibody failures, all attempts at replication were successful. For yeast assays, once assay conditions were defined, experiments were independently replicated successfully at least 3 times and representative data are shown. For mouse protein assays, protein lysates of at least 4 mice for each line and multiple organs were tested to confirm lack of NEMF expression. All attempts were successful.
Randomization	Organisms were allocated into groups based on the genotyping results from PCR-based and Sanger sequencing for the NEMF variant of interest (D106*, R86S or R487G), mice were determined to be Wildtype, heterozygous or homozygous for the variant.
Blinding	For analysis of pathophysiology and behavioral measures assessor was genotype blind (i.e. NMJ occupancy, axon counts, body weights, oxygen saturation, wire hang). For assessment of protein levels (western blot) assessor was not blinded, as genotypes needed to be in specific order. Blinding was not relevant for Yeast experiments (Figure 4 and Supplementary Figure 10), as they were controlled across samples using protein concentrations and internal controls and investigator bias is not considered to contribute to the data.

Reporting for specific materials, systems and methods

We require information from authors about some types of materials, experimental systems and methods used in many studies. Here, indicate whether each material, system or method listed is relevant to your study. If you are not sure if a list item applies to your research, read the appropriate section before selecting a response.

Materials & experimental systems

n/a	Involved in the study	
☐	☒	Antibodies
☒	☐	Eukaryotic cell lines
☒	☐	Palaeontology
☐	☒	Animals and other organisms
☐	☒	Human research participants
☒	☐	Clinical data

Methods

n/a	Involved in the study	
☒	☐	ChIP-seq
☒	☐	Flow cytometry
☒	☐	MRI-based neuroimaging

Antibodies

Antibodies used	anti-2H3 (Developmental Studies Hybridoma Bank, 2H3, AB_2314897, 1-12-17 and 2-22-18), anti-SV2 (Developmental Studies Hybridoma Bank, SV2, AB_2315387, 9-20-18), Alexa-Fluor 488 goat anti-mouse IgG1 (Invitrogen, catalog # A-21121, RRID AB_2535764, lot#2040297), α-bungarotoxin conjugated with 441 Alexa-Fluor 594(Invitrogen, catalog #B13423, lot#1938422), anti-NEMF, (Proteintech, anti-SDCCAG1, 11840-1-AP,1:1000), anti-GAPDH (Cell Signaling, Cat #2118, clone 14C10, 1:20000), Anti-FLAG tag (Sigma Clone M2 1:1000), Anti-HA (Roche Clone 3F10 1:1000), Anti-GFP (Roche Clone 7.1 and 13.1, 1:1000), Anti-PGK1 (Invitrogen Clone 22C5D8 1:10000), Anti-Sis1 (E. Craig lab, 1:10,000-20,000).
Validation	anti-2H3, anti-SV2, Alexa-Flour 488 and a-BTX 594 were used for immunoflourescent staining of the pre and post-synaptic portions of the neuromuscular junctions as previously described in Bogdanik, et.al. 2013. anti-NEMF is validated on the manufacture's website with both A549 and HEK-293 cells (http://www.ptgcn.com/products/SDCCAG1-Antibody-11840-1-AP.htm), as well as with our own NEMF-null model (NEMF-D106*/D106*) in Figure 3 a. anti-GAPDH has also been validated with various cell lines (https://www.cellsignal.com/products/primary-antibodies/gapdh-14c10-rabbit-mab/2118). Anti-FLAG was validated and optimized for single banded detection of FLAG fusion proteins in mammalian, plant, and bacterial expression systems (https://www.sigmaaldrich.com/catalog/product/sigma/f1804?lang=en&region=US). Anti-HA was function validated in

western blot by manufacture (https://www.sigmaaldrich.com/catalog/product/roche/roahaha?lang=en&region=US&gclid=EAIaIQobChMIyPj90Oa46gIVFqqWCh017Q_OEAAYASAAEgJB-vD_BwE). Anti-GFP was tested for functionality and purity relative to a reference standard by manufacturer. (https://www.sigmaaldrich.com/catalog/product/roche/11814460001?lang=en&region=US). We have consistently tested FLAG, HA and GFP antibodies in protein lysates that do contain tag-fused protein and lysates that does not to confirm specificity to the tag. Anti-PGK1 Manufacturer recommends use in the application used in the study. They also site more then 100 publication that utilize this antibody for Immunoblotting. (https://www.thermofisher.com/antibody/product/PGK1-Antibody-clone-22C5D8-Monoclonal/459250). We have also consistently used this antibody reliably for Yeast extracts such as in Yonashiro, R. et al Elife 5, 1–16 (2016). Anti-SIS1 was previously validated in Yan, W. & Craig, E. A. Mol. Cell. Biol. 19, 7751–7758 (1999).

Animals and other organisms

Policy information about studies involving animals; ARRIVE guidelines recommended for reporting animal research

Laboratory animals

Mice were bred and maintained under standard conditions. Mice were maintained on a C57BL/6J background. Tissues from mice were harvested at times indicated in methods. For each Mus musculus strain were maintained on a C57BL/6J background carrying the strain indicated variant (Nemf-R86S, Nemf-R487G and Nemf-D106*) and roughly equal males and females were observed per time-point per genotype (wildtype, heterozygous and homozygous). Mice were maintained in the JAX research animal facility room under the standard described conditions until tissue collection at the time-points specified. For NEMF-R487G (WT, HET, HOMs) were harvested at (2W, 8W, 55W, 1.5-2years) for NEMF-R86S (WT, HET, HOM) were aged to and harvested at (2W, 16-18 days, 8W) and for NEMF-D106* (WT, HET, HOM) were aged to and harvested at (9-11 days (end of life for HOM)).

Wild animals

This study did not involve wild animals.

Field-collected samples

This study did not involve field collected samples.

Ethics oversight

All mouse husbandry and procedures were reviewed and approved by the respective Institutional Animal Care and Use Committees at The Jackson Laboratory and Scripps Research, and were carried out according to the NIH Guide for Care and Use of Laboratory Animals.

Note that full information on the approval of the study protocol must also be provided in the manuscript.

Human research participants

Policy information about studies involving human research participants

Population characteristics

Human research participants are cases that sought clinical assessment of neuromuscular and/or intellectual disability and sought a genetic diagnosis. The characteristics of the human research participants can be found in Table 1, Age of onset range from 1.5 to 17 years of age, 6 males and 3 females, genotypic information is outlined in the Figure 5 pedigrees. Participants were not assigned to treatment categories.

Recruitment

The patients were recruited through the use of the GeneMatcher tool, wherein, the research patients had attended a clinic for assessment of phenotypes (i.e. neuromuscular deficits and/or intellectual disability) and clinicians requested Whole Exome or Genome sequencing in order to identify possible genetic contributions. Clinicians with the NEMF variant hits uploaded the phenotype/gene of interest into GeneMatcher where collaborative matches were made. Self-selection biases would be that these patients were those who warranted clinical assessment.

Ethics oversight

Approval for the studies have been from the following: The Institutional Review Board (IRB) at Baylor College of Medicine (protocol number H-29697); considered IRB exempt at the University of South Florida and Mission Fullerton Genetics Center; The Australian Genomics Neuromuscular Disorders ethics committee approval from Melbourne Health (HREC/16/MH251); Approval from the ethics commission of Otto-von-Guericke-Universität and the Medizinischen Fakultät at Universitätsklinikum Magdeburg A.ö.R.; the IRB at The University college London; the IRB at King Fahad Medical City (IRB#19-512).

Note that full information on the approval of the study protocol must also be provided in the manuscript.
