# Supplementary material for: NEMF mutations that impair ribosome-associated quality control are associated with neuromuscular disease
Source: Nat Commun. 2020 Sep 15;11:4625. doi: 10.1038/s41467-020-18327-6 (PMC7494853; doi:10.1038/s41467-020-18327-6)
Supplement: Supplementary file 4 — Peer Review File [file 41467_2020_18327_MOESM4_ESM.docx]

Peer Review Information

**Journal:** Nature Communications

**Manuscript title:** NEMF mutations that impair ribosome-associated quality control are associated with neuromuscular disease

**Corresponding author name(s):** Dr Gregory Cox and Claudio A.P. Joazeiro

**Editorial notes:**

**none**

**Reviewer comments & decisions:**

**Reviewer comments, first version:**

Reviewer #1 (Remarks to the Author):

Defects in ribosome-associated quality control (RQC) are widely considered to be a cause of neurodegeneration, but this hypothesis is primarily based on a single report concerning the consequences of mutation in the E3 ubiquitin ligase Listerin in mice. There is therefore great interest in identifying and characterizing additional genetic defects in this and other components of the RQC pathway that are associated with neurodegenerative disease in humans.

Here, Martin et al. present a comprehensive study of the consequences of mutation of the RQC factor NEMF in mice (Rqc2 in yeast), complemented by the identification of mutations in this factor that are associated with neurodegenerative disease in humans. Following the splitting of stalled 80S ribosomes, Rqc2 facilitates the proteasomal degradation of nascent peptides associated with the 60S subunits by catalyzing their C-terminal extension with non-templated Alanine and Threonine tails. ENU mutagenesis of mice yielded independent homozygous R86S and R487G substitutions in NEMF that were associated with motor and growth defects, neurogenic atrophy, premature lethality and an early-onset age-dependent neurodegenerative phenotype. A CRISPR-generated NEMF null mutant caused by premature termination of NEMF translation was associated with more severe symptoms. Equivalents of the R86S and R487G substitutions made in the homologous yeast factor Rqc2 were predicted to affect its catalytic activity and were found to result in partial defects in CAT-tailing. Although CAT-tailing has not conclusively been observed in humans, these observations suggest that it may be an important aspect of NEMF’s function in human RQC, and that defects in this process can ultimately result in neurodegeneration.

Genome-wide association studies have previously implicated mutations in NEMF as a potential cause of developmental delay and intellectual disability (Ref. 25) and here, Martin et al. identified 6 individuals with mutations in NEMF that were associated with neuropathy, motor disfunction, speech delay and intellectual disability.

In conclusion, this clearly presented manuscript integrates data obtained using a variety of experimental approaches to implicate a second RQC-associated factor as a cause of neurodegeneration in mammals, including humans. It will be of broad general interest and merits publication


Reviewer #2 (Remarks to the Author):

This is a very nice description of three novel mouse models, and then a set of human families, that highlight the importance of the NEMF gene and ribosome quality control in neurodegenerative disease. This will be an important first report for looking further at disease mechanism and the link between aberrant ribosome-associated quality control (RQC), aberrant translation, and neurodegeneration – and here the resource (the mice) for understanding mechanism is presented and defined. This work builds on previous investigations of mutation in the E3 ligase, Listerin.

A few comments:
• The ENU models are well-presented, but a criticism – mainly from labs not working with ENU mutants – is of potential mutations outside the gene, but closely linked. Can the authors put in a statement, perhaps in methods, about backcrossing/sequencing/having 3 independent mouse lines/etc, to allay such fears.
• The authors must be crystal clear, for every piece of data presented about the sex of the mice used and the numbers. If they combine males and females this must be justified (as for D106* behaviour). For example, which sex data is used for Figure 1? And on.
• The NMJ staining was rather hard to make out on my computer/hardcopy but that may be unavoidable.
• Figure 2 and 3 have been muddled in the text e.g. the section on distal and proximal motor axons refers to Figs 3n,o,s, which is obviously Figure 2.
• Can the authors supply a brief comment explicitly ruling out overt neurodevelopmental phenotypes i.e. that what we are seeing is neurodegeneration?
• Do the authors have any data on heterozygous mouse phenotype (or lack of phenotype)?
• Similarly, do they have any brain histology, it would be fascinating to see this, in respect to the human families, but is obviously a whole other large study and not necessarily for this paper.


Reviewer #3 (Remarks to the Author):


This original and impressive manuscript establishes the molecular role of the NEMF protein in mammalian cells and its contribution to inherited neurological disease in man. It constitutes a tour d’force that presents phenotypic and molecular data from 3 mouse alleles, functional assays in yeast, and five human patients with variants that are deleterious and appear to be pathogenic. The data is high quality and convincingly supports the authors’ interpretations. Overall the manuscript provides evidence for the novel finding that “CAT-tailing” of nascent chains on stalled ribosomes is an important protective mechanism for turnover of incomplete proteins in mammalian neurons. The paper is very well written and complex data is clearly described. The following minor suggestions are recommended for clarification of certain points in the text:

summary line 47: delete ‘with’. and add ‘by addition of’

page 3 line 63: delete ‘next’

page 4 line 110: by 8 weeks INSERT “surviving” R86S mice....

page 5. line 154-156: I do not see a Figure 3t, is this an error?.

page 6 line 191: begin new sentence after refs 11,13. “The R86S mutation is located in the vecinity of the universally-conserved.....”

page 7 line 220: after “lane 1” insert “(anti-GFP)

Page 7 paragraphs 2 and 3: add description of lanes expressing D99Y

page 8 line245; after “reporter levels” insert “(anti-HA)”

Page 11 line 375 methods: explain the lesion in the R487 mouse and the relationship between mutant and wildtype forward primers.


Reviewer #4 (Remarks to the Author):

The authors provide convincing evidence through the study of 3 mouse models that loss-of-function mutations of NEMF leads to neurodegeneration through defective ribosome quality control and identified several children with several neurological disorders caused by severe, biallelic NEMF mutations.

Comments

1. Patient USA3 shows a far milder clinical phenotype as compared to the other patients associated to compound heterozygous NEMF variants, one of them a deep intronic variant. The authors argue based on bioinformatic predictions that the intronic variant results in missplicing. However, they do not provide experimental evidence for missplicing. The authors need to provide this evidence

2. Figure 2a-d: Please could the authors confirm that all images have been taken at the same magnification? A scale bar of 100μm is given, but the images between WT and corresponding animal model look to be very different in size.


3. Again with fig 2a-d it would be helpful to have some images much more zoomed in or at a higher magnification. While there may be a few signs of atrophy this is not “extensive” as the authors describe. In addition, if this was neurogenic we would expect to see different fibre sizes. It would be important to perform some quantification of these fibre sizes and maybe show some fibre type grouping (MHC staining) to demonstrate that this is in fact an extensive neurogenic atrophy and not a generalized reduction in fibre size maybe attributable to being less active.

4. Figures 2e-i: Please could the authors provide some images at higher magnification or zoomed in, as it is currently very difficult to see the fragmentation and denervation features they refer to. It would also appear in the WT mice at 8 weeks there is no α-bungarotoxin staining as the 4 NMJS in Fig 2E are all green. Is there any reason for this? It is difficult to see how these NMJs were recorded as being innervated (Fig 2j) when there is no α-bungarotoxin staining, if there was overlap these should be yellow.


5. Not only is there denervation in the R86S NMJs at 8 weeks but they also appear more diffuse and less structured than both the WT and R487G NMJs. Although the fragmentation the authors refer to is not clear, was the fragmentation quantified? How was this defined?

6. Figure 1c and Supp figure 1: Do they have any more weight data for the R487G and WT mice? A jump from 8 weeks to 55 is rather large, especially since it does not appear that the growth curves have plateaued at 8 weeks. In addition the direction of the line at week 55 suggests many of the mice were still on an upward trajectory. A couple more data points after 8 weeks and maybe before the 55 week mark would be useful.


7. Figure 1d: Do the authors have any idea or hypothesis as to why the animals die?

8. Pg 5 line 140 – should this say figure 2 not 3? Also lines 142, 143, 154, 155, 156?


9. Could the authors be consistent in the order they present data and images, for example Fig 2K-R the images are presented WY/R487G/R86S, but then in the graphs (fig 2S&T) the data is given WT/R86S/R487G. Figure 2A-D is presented WT/R86S, while Fig 2E-G is presented WT/R487G/R86S.

10. Could the authors include some images associated with Fig 2t, and sup fig 4e?


11. Please could the authors clarify the difference between Supp Fig 6b showing a western blot of brains of 10 day old WT, Nemf D106* homo- and het mice and Figure 3a showing western blot analysis of brains of 10 day old WT, Nemf D106* homo-and het mice? In the supplementary figure there is a clear non-specific band indicated with an * however this seems to have disappeared in figure 3a.

12. The authors state that D106* animals have greater denervation than WT mice (sup fig 6e). Thought the associated image (Supp 6d) clearly shows that there are plenty of innervated nerves stained with neurofilament, however many of these NMJs do not show SV2 labelling. Do the authors contend that these NMJs are denervated or is there another mechanism causing the loss of SV2 labelling? The loss of myelination (fig 3e) could result in a thinning of these axons, but the NMJs still look to be innervated.


13. Could the authors state if they checked for normality of distribution? How did they deal with any outliers?

**Author rebuttal, first version:**

**Reviewer #1 (Remarks to the Author):**
Defects in ribosome-associated quality control (RQC) are widely considered to be a cause of neurodegeneration, but this hypothesis is primarily based on a single report concerning the consequences of mutation in the E3 ubiquitin ligase Listerin in mice. There is therefore great interest in identifying and characterizing additional genetic defects in this and other components of the RQC pathway that are associated with neurodegenerative disease in humans.

Here, Martin et al. present a comprehensive study of the consequences of mutation of the RQC factor NEMF in mice (Rqc2 in yeast), complemented by the identification of mutations in this factor that are associated with neurodegenerative disease in humans. Following the splitting of stalled 80S ribosomes, Rqc2 facilitates the proteasomal degradation of nascent peptides associated with the 60S subunits by catalyzing their C-terminal extension with non-templated Alanine and Threonine tails. ENU mutagenesis of mice yielded independent homozygous R86S and R487G substitutions in NEMF that were associated with motor and growth defects, neurogenic atrophy, premature lethality and an early-onset age-dependent neurodegenerative phenotype. A CRISPR-generated NEMF null mutant caused by premature termination of NEMF translation was associated with more severe symptoms. Equivalents of the R86S and R487G substitutions made in the homologous yeast factor Rqc2 were predicted to affect its catalytic
activity and were found to result in partial defects in CAT-tailing. Although CAT-tailing has not conclusively been observed in humans, these observations suggest that it may be an important aspect of NEMF’s function in human RQC, and that defects in this process can ultimately result in neurodegeneration.

Genome-wide association studies have previously implicated mutations in NEMF as a potential cause of developmental delay and intellectual disability (Ref. 25) and here, Martin et al. identified 6 individuals with mutations in NEMF that were associated with neuropathy, motor disfunction, speech delay and intellectual disability.

In conclusion, this clearly presented manuscript integrates data obtained using a variety of experimental approaches to implicate a second RQC-associated factor as a cause of neurodegeneration in mammals, including humans. It will be of broad general interest and merits publication

We are grateful to the reviewer for the overview that nicely captures the essence of our work, and the supportive comments.

**Reviewer #2 (Remarks to the Author):**
This is a very nice description of three novel mouse models, and then a set of human families, that highlight the importance of the NEMF gene and ribosome quality control in neurodegenerative disease. This will be an important first report for looking further at disease mechanism and the link between aberrant ribosome-associated quality control (RQC), aberrant translation, and neurodegeneration – and here the resource (the mice) for understanding mechanism is presented and defined. This work builds on previous investigations of mutation in the E3 ligase, Listerin.

A few comments:

• The ENU models are well-presented, but a criticism – mainly from labs not working with ENU mutants – is of potential mutations outside the gene, but closely linked. Can the authors put in a statement, perhaps in methods, about backcrossing/sequencing/having 3 independent mouse lines/etc, to allay such fears.

This is an excellent point. We have indeed considered the possibility that additional ENU-induced mutations linked to NEMF contribute to the phenotypes we report. We do not think that is the case for several reasons: (1) the NEMF R86S allele was mapped to a 4.6 Mb interval in an F2 intercross (Chr12:65486719-70085250 bp, GRCm38 mouse genome assembly) and, even though the region was not exhaustively sequenced, the only exonic variant detected among 15 candidate genes analyzed was in the *Nemf* gene; (2) the fact that a second independent ENU-induced mutation (NEMF R487G) was mapped to the same region of Chr 12 and produced affected offspring in an allelism test with the NEMF-R86S allele suggested the observed phenotype is being driven by the NEMF variants alone; (3) both ENU alleles were backcrossed to C57BL/6J for at least 10 generations to eliminate any unlinked and distantly linked ENU-induced mutations as potential genetic confounds; (4) finally, the independent CRISPR-induced NEMF D106* allele also produced affected recessive offspring . We have included this information in the methods section.

• The authors must be crystal clear, for every piece of data presented about the sex of the mice used and the numbers. If they combine males and females this must be justified (as for D106* behaviour). For example, which sex data is used for Figure 1?

To address the reviewer’s comment, we added qualifying statements to phenotyping assays where sex was combined (i.e. wire hang, myelinated axon counts, NMJ assessments, oxygen saturation, growth curve for D106* mice): “It was determined that there were no sex differences in phenotype and therefore the data was presented as mixed sex, with similar numbers of males and females.”

• The NMJ staining was rather hard to make out on my computer/hardcopy but that may be unavoidable.

To address the reviewer’s comment, we now provide representative images with enhanced brightness and contrast that will allow readers to better visualize the features in reference both on the computer screen and in hardcopy.

• Figure 2 and 3 have been muddled in the text e.g. the section on distal and proximal motor axons refers to Figs 3n,o,s, which is obviously Figure 2.

We thank the reviewer for pointing out the error, which has now been fixed.

• Can the authors supply a brief comment explicitly ruling out overt neurodevelopmental phenotypes i.e. that what we are seeing is neurodegeneration?

In 14-day and younger animals, the results for, e.g., myelinated axons and body weight, were comparable between wildtype mice and the R86S and R487G mutants. We interpret these observations as indicating that it is unlikely that we are dealing with overt neurodevelopmental phenotypes.

• Do the authors have any data on heterozygous mouse phenotype (or lack of phenotype)?

Yes, this data is presented in supplementary figures 2 and 3. The phenotypes of heterozygous mice were comparable to wild type mice with regard to wire hang, growth curves, and lifespan. We have now also included quantification of femoral motor and L4 ventral root axons from 1.5-year and older mice in supplementary figure 3. Even in these later time points no differences were observed for the heterozygous mice.

• Similarly, do they have any brain histology, it would be fascinating to see this, in respect to the human families, but is obviously a whole other large study and not necessarily for this paper.

We agree that brain histology would be interesting to analyze, and have plans to do so in future work with the mouse models. For the few patients whose MRI or CT brain imaging data is available, no consistent phenotypes have been observed so far.

We thank the reviewer for these excellent critiques that have helped improve the quality of our manuscript.

**Reviewer #3 (Remarks to the Author):**
This original and impressive manuscript establishes the molecular role of the NEMF protein in mammalian cells and its contribution to inherited neurological disease in man. It constitutes a tour d’force that presents phenotypic and molecular data from 3 mouse alleles, functional assays in yeast, and five human patients with variants that are deleterious and appear to be pathogenic. The data is high quality and convincingly supports the authors’ interpretations. Overall the manuscript provides evidence for the novel finding that “CAT-tailing” of nascent chains on stalled ribosomes is an important protective mechanism for turnover of incomplete proteins in mammalian neurons. The paper is very well written and complex data is clearly described. The following minor suggestions are recommended for clarification of certain points in the text:

summary line 47: delete ‘with’. and add ‘by addition of’

The manuscript has been revised per the reviewer’s suggestion.

page 3 line 63: delete ‘next’

The manuscript has been revised per the reviewer’s suggestion.

page 4 line 110: by 8 weeks INSERT “surviving” R86S mice....

We thank the reviewer for this suggestion, which will certainly help clarify that the 8-week old R86S mice analyzed represent a subset of animals that survived beyond the median life span.

page 5. line 154-156: I do not see a Figure 3t, is this an error?.

We thank the reviewer for pointing the error out. The manuscript has been revised accordingly.

page 6 line 191: begin new sentence after refs 11,13. “The R86S mutation is located in the vicinity of the universally-conserved.....”

The manuscript has been revised per the reviewer’s suggestion.

page 7 line 220: after “lane 1” insert “(anti-GFP)

The manuscript has been revised per the reviewer’s suggestion.

Page 7 paragraphs 2 and 3: add description of lanes expressing D99Y

The manuscript has been revised per the reviewer’s suggestion.

page 8 line245; after “reporter levels” insert “(anti-HA)”

The manuscript has been revised per the reviewer’s suggestion.

Page 11 line 375 methods: explain the lesion in the R487 mouse and the relationship between mutant and wildtype forward primers.

We thank the reviewer for pointing the error out. The mutant forward primer sequence was incorrectly labelled as the common reverse primer and *vice* *versa*. We have now fixed the error.

We thank the reviewer for these excellent critiques that have helped improve the quality of our manuscript.

**Reviewer #4 (Remarks to the Author):**
The authors provide convincing evidence through the study of 3 mouse models that loss-of-function mutations of NEMF leads to neurodegeneration through defective ribosome quality control and identified several children with several neurological disorders caused by severe, biallelic NEMF mutations.

Comments

1. Patient USA3 shows a far milder clinical phenotype as compared to the other patients associated to compound heterozygous NEMF variants, one of them a deep intronic variant. The authors argue based on bioinformatic predictions that the intronic variant results in missplicing. However, they do not provide experimental evidence for missplicing. The authors need to provide this evidence

Patient USA3 indeed has a milder phenotype. Further work using ddPCR has revealed that the deep intronic variant is in a *cis* position relative to the *de novo* variant. This variant has now been ruled as a variant of unknown significance (VOUS) and is unlikely to be a contributing factor to the phenotype. Accordingly, we have revised the manuscript to indicate that the USA3 patient potentially reflects a dominantly inherited or dominant-negative case.

2. Figure 2a-d: Please could the authors confirm that all images have been taken at the same magnification? A scale bar of 100μm is given, but the images between WT and corresponding animal model look to be very different in size.

We thank the reviewer for pointing the error out. The scale bar for the 16-day old mice (Fig 2 a,b) was indeed missing. Although the muscles in the younger animals shown are certainly smaller, there is indeed a magnification difference between the panels. This figure has now been edited and images using the correct scale bar are shown in Supplementary Fig.2.

3. Again with fig 2a-d it would be helpful to have some images much more zoomed in or at a higher magnification. While there may be a few signs of atrophy this is not “extensive” as the authors describe. In addition, if this was neurogenic we would expect to see different fibre sizes. It would be important to perform some quantification of these fibre sizes and maybe show some fibre type grouping (MHC staining) to demonstrate that this is in fact an extensive neurogenic atrophy and not a generalized reduction in fibre size maybe attributable to being less active.

Per the reviewer’s suggestion, we have updated the images to provide better representation of the different fiber sizes that we observe. The images now cover a larger area of the MG muscle. An additional image is now presented for the 55-week old R487G mutant in Supp. Fig2 to help make this point.

4. Figures 2e-i: Please could the authors provide some images at higher magnification or zoomed in, as it is currently very difficult to see the fragmentation and denervation features they refer to. It would also appear in the WT mice at 8 weeks there is no α-bungarotoxin staining as the 4 NMJS in Fig 2E are all green. Is there any reason for this? It is difficult to see how these NMJs were recorded as being innervated (Fig 2j) when there is no α-bungarotoxin staining, if there was overlap these should be yellow.

We thank the reviewer for the suggestion. The figures now have an inset showing an enlargement of a portion of the image, allowing readers to more clearly see fragmentation and denervation features.

The 8-week old mice do show α-bungarotoxin staining in the red channel, which just needed to be increased in brightness and contrast to allow better visualization of the post-synaptic terminals and overlap of the NMJ. This is clearly visualized on both the confocal and stereoscope in the red channel. Improved images have been provided to emphasize this point.

5. Not only is there denervation in the R86S NMJs at 8 weeks but they also appear more diffuse and less structured than both the WT and R487G NMJs. Although the fragmentation the authors refer to is not clear, was the fragmentation quantified? How was this defined?

The diffuse and less structured nature of the post-synaptic terminals represent the final stages of fragmentation, most notable in R86S 8W (Fig 2c) and R487G 55W (Fig2e). This fragmentation was not quantified, but post-synaptic regions that were visible were defined as denervated as there was no overlapping of pre-synaptic SV2 and 2H3 immunostaining.

6. Figure 1c and Supp figure 1: Do they have any more weight data for the R487G and WT mice? A jump from 8 weeks to 55 is rather large, especially since it does not appear that the growth curves have plateaued at 8 weeks. In addition the direction of the line at week 55 suggests many of the mice were still on an upward trajectory. A couple more data points after 8 weeks and maybe before the 55 week mark would be useful.

We thank the reviewer for making this excellent point. We have now included extended data for the 20-week old R487G mutant and wildtype mice, bridging the gap between 8 and 55 weeks.

7. Figure 1d: Do the authors have any idea or hypothesis as to why the animals die?

21-day old R86S homozygous mice are still very small and it isn’t clear if their death is due to feeding difficulty leading to nutritional deficiency, to potential respiratory defects, or to other reasons. Although the phrenic nerves are impacted in the NEMF-null model at end of life, at end of life in the R86S we do not see a difference in phrenic nerve myelinated axon count. Future work will investigate potential defects in respiratory function as a lethal factor.

8. Pg 5 line 140 – should this say figure 2 not 3? Also lines 142, 143, 154, 155, 156?

We thank the reviewer for pointing the error out. It has now been corrected.

9. Could the authors be consistent in the order they present data and images, for example Fig 2K-R the images are presented WY/R487G/R86S, but then in the graphs (fig 2S&T) the data is given WT/R86S/R487G. Figure 2A-D is presented WT/R86S, while Fig 2E-G is presented WT/R487G/R86S.

We thank the reviewer for the comment. We have now reworked figure 2 to make the order of the images and data more consistent.

10. Could the authors include some images associated with Fig 2t, and sup fig 4e?
Per the reviewer’s request, representative images of the L4 Ventral roots associated with figure 2t and supplemental figure 4e are now available in supplemental figure 3.

11. Please could the authors clarify the difference between Supp Fig 6b showing a western blot of brains of 10 day old WT, Nemf D106* homo- and het mice and Figure 3a showing western blot analysis of brains of 10 day old WT, Nemf D106* homo-and het mice? In the supplementary figure there is a clear non-specific band indicated with an * however this seems to have disappeared in figure 3a.

The reason why supplementary figure 6b shows a non-specific band, while Fig.3a does not is that those blots were generated using different antibodies. Since the overall data is indeed redundant between those two figures, we have revised the manuscript such that only the panel in figure 3 is now shown.

12. The authors state that D106* animals have greater denervation than WT mice (sup fig 6e). Though the associated image (Supp 6d) clearly shows that there are plenty of innervated nerves stained with neurofilament, however many of these NMJs do not show SV2 labelling. Do the authors contend that these NMJs are denervated or is there another mechanism causing the loss of SV2 labelling? The loss of myelination (fig 3e) could result in a thinning of these axons, but the NMJs still look to be innervated.
We interpret the results as indicating that the D106* mutant mice have a greater extent of denervation than wildtype mice. The image in supp figure 6 shows clear post-synaptic and 2H3/SV2 labelling in the wildtype image, while the D106* mutant image only shows the presynaptic nerve but not the end-plate. For this experiment, muscle tissue was incubated in the same master mix of primary antibodies and therefore the anti-SV2 antibody was functional in both as evident by staining in the wildtype.

More appropriate representative images have now been provided, as well as two images for the D106* mutant mice to show that while some of the nerve is still present, the endplates have started to die back.

13. Could the authors state if they checked for normality of distribution? How did they deal with any outliers?

We thank the reviewer for the comment. We did check the data for normality of distribution using a Shapiro-Wilk normality test in Prism 7. We did not remove outliers from the data. A statement in our statistical method section has been added to reflect this.

We thank all reviewers for their thoughtful and thorough comments and hope they will find our responses satisfactory and the revised manuscript appropriate for publication

**Reviewer comments, second version:**

REVIEWERS' COMMENTS:

Reviewer #2 (Remarks to the Author):

I have no further comments.


Reviewer #4 (Remarks to the Author):

The authors have addressed all of my comments and improved the figures substantially.

**Author rebuttal, second version:**

**Reviewer comments, third version:**

**Author rebuttal, third version:**
